# Bacterial symbionts support larval sap feeding and adult folivory in (semi-)aquatic reed beetles

**DOI:** 10.1038/s41467-020-16687-7

**Published:** 2020-06-11

**Authors:** Frank Reis, Roy Kirsch, Yannick Pauchet, Eugen Bauer, Lisa Carolin Bilz, Kayoko Fukumori, Takema Fukatsu, Gregor Kölsch, Martin Kaltenpoth

**Affiliations:** 1 Evolutionary Ecology, Institute for Organismic and Molecular Evolution (iomE), Johannes Gutenberg University, Hanns-Dieter-Hüsch-Weg 15, 55128 Mainz, Germany; 2 Department of Entomology, Max Planck Institute for Chemical Ecology, Hans-Knöll-Str. 8, 07745 Jena, Germany; 3 Bioproduction Research Institute, National Institute of Advanced Industrial Science and Technology, Tsukuba, 305-8566 Japan; 4 Molekulare Evolutionsbiologie, Institut für Zoologie, Universität Hamburg, Martin-Luther-King-Platz 3, 20146 Hamburg, Germany; 5 Present Address: Plant Evolutionary Ecology, Institute of Evolution and Ecology, University of Tübingen, Auf der Morgenstelle 5, 72076 Tübingen, Germany; 6 Present Address: Maasen 6, 24107 Kiel, Germany

**Keywords:** Evolutionary ecology, Microbial ecology, Environmental microbiology, Entomology

## Abstract

Symbiotic microbes can enable their host to access untapped nutritional resources but may also constrain niche space by promoting specialization. Here, we reconstruct functional changes in the evolutionary history of the symbiosis between a group of (semi-)aquatic herbivorous insects and mutualistic bacteria. Sequencing the symbiont genomes across 26 species of reed beetles (Chrysomelidae, Donaciinae) spanning four genera indicates that the genome-eroded mutualists provide life stage-specific benefits to larvae and adults, respectively. In the plant sap-feeding larvae, the symbionts are inferred to synthesize most of the essential amino acids as well as the B vitamin riboflavin. The adult reed beetles’ folivory is likely supported by symbiont-encoded pectinases that complement the host-encoded set of cellulases, as revealed by transcriptome sequencing. However, mapping the occurrence of the symbionts’ pectinase genes and the hosts’ food plant preferences onto the beetles’ phylogeny reveals multiple independent losses of pectinase genes in lineages that switched to feeding on pectin-poor plants, presumably constraining their hosts’ subsequent adaptive potential.

## Abstract (web summary)

Symbiotic microbes in insects can enable their hosts to access untapped nutritional resources. Here, the authors show that symbiotic bacteria in reed beetles can provide essential amino acids to sap-feeding larvae and help leaf-feeding adults to degrade pectin, respectively.

## Introduction

Microbial symbionts are pervasive in herbivorous insects and often aid in alleviating the challenges associated with a plant-based feeding ecology^1^. In particular, symbiotic microorganisms can supplement their host’s limited diet by provisioning essential amino acids or vitamins, or provide enzymes for the digestion of fastidious polymers or the detoxification of noxious plant secondary metabolites^1–3^. As such, symbiotic associations with beneficial microbes have facilitated evolutionary transitions to ecological niches that would otherwise have been inaccessible to insects due to their limited metabolic capabilities^4^. In particular, the exploitation of plant sap and the ensuing diversification in the speciose insect order Hemiptera was dependent on the presence of microbial symbionts compensating for the scarcity of essential amino acids and vitamins in the diet^4,5^. Similarly, wood-feeding termites^6^, passalid beetles^7^, and the leaf-chewing tortoise beetle^8^ are assisted by symbiotic microbes for the break-down of the major components of plant cell walls that present rich but enzymatically challenging sources of carbon and energy^9^. Despite an increasing number of studies demonstrating the functional importance of microbial symbionts for the nutrition of herbivorous insects, however, detailed knowledge about the evolutionary origins of such symbioses and their ecological implications remains confined to a few well-investigated insect taxa^10^. Furthermore, functional consequences of subsequent evolutionary changes in the symbionts’ metabolic potential remain poorly investigated, limiting our understanding of how microbial symbionts can expand, but also constrain, ecological niche space of their host insects.

Reed beetles (Coleoptera, Chrysomelidae, Donaciinae) represent an ecologically unusual group of leaf beetles, comprising approximately 165 aquatic and semi-aquatic species^11^. Like their chrysomelid relatives, adult reed beetles are leaf-feeders that are, however, confined to aquatic host plants, mostly within the orders Poales, Nymphaeales, and Alismatales, which serve as feeding and oviposition sites^11,12^. Uniquely, the larvae of Donaciinae attach to their host plant’s submerged roots, feeding on the sap of the plant and obtaining oxygen from the intercellular spaces through specialized hook-like organs located at the tip of the abdomen^13,14^. Thus, as opposed to most other chrysomelids, the sources of nutrition differ severely between adults and larvae of the same species, likely confronting the beetles with the nutritional challenges of both sap-feeding and leaf-chewing insects. A potential source of nutritional supplements are microbial symbionts, and adult reed beetle females are known to harbor symbiotic bacteria intracellularly and extracellularly in modified Malpighian tubules and transmit them to the eggs within a specialized jelly coat^15–17^. In hatching larvae, the symbionts quickly colonize the epithelial cells of midgut-associated organs, from where they transition to the Malpighian tubules in late larval stages^15^. Experimental manipulation of the symbiosis revealed that aposymbiotic larvae are unable to complete their cocoon for pupation and metamorphosis, but the metabolic contributions of the symbionts remained enigmatic^18^.

Here, we investigated the contribution of the bacterial symbionts to the feeding ecology of larval and adult Donaciinae beetles by using comparative genomics of the symbionts across 26 host species, in combination with host transcriptomics and functional assays to elucidate the potential of hosts and symbionts to break down plant cell wall components. Our results reveal that the symbiont genomes are streamlined for essential amino acid and riboflavin supplementation to complement the larval plant sap-based diet, providing an explanation for the inability of aposymbiotic larvae to produce the proteinaceous cocoon. In addition, adult folivory is likely supported by two symbiont-encoded pectinases, but only in the host species feeding on Nymphaeales or Alismatales, whereas the taxa feeding on pectin-poor Poales host plants secondarily lost the pectinases from the symbiont genomes. Hence, our study provides evidence for (i) the convergent evolution of symbiont-mediated nutritional supplementation in a sap-feeding taxon outside of the well-studied Hemiptera, (ii) a case of two different life-stage specific benefits that symbionts confer on larvae (supplementation of essential amino acids and riboflavin) and adults (support of digestion by provisioning of pectinases), respectively, and (iii) a scenario in which the loss of symbiont-encoded functions after host plant switching constrained the beetle’s adaptive potential and confined it to a particular group of host plants.

## Results

### Reed beetle symbiont genomes are eroded and syntenic

**Fig. 1:**
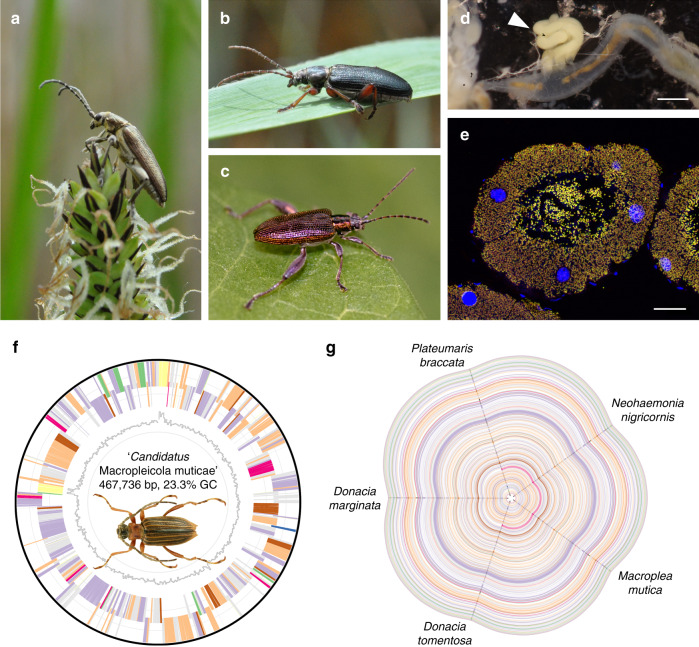
Reed beetle symbionts show strongly reduced and perfectly syntenic genomes. **a**–**c** Representative images of Donaciinae beetles, **a** *Donacia thalassina*, **b** *Plateumaris braccata*, **c** *Donacia versicolorea* (picture kindly provided by Rebekka Janke). **d** Localization of symbiotic organs (white arrowhead) at the midgut/hindgut junction. Scale bar 0.5 mm. **e** Fluorescence in situ hybridization micrograph showing a cross-section of the symbiotic organs of a female *Donacia vulgaris*. Fluorescently labeled symbionts (yellow) are visible in the cells and the lumen of the enlarged Malpighian tubules. General DNA counterstaining was done with DAPI (blue). Scale bar 40 µm. **f** Genome of “*Candidatus* Macropleicola muticae”, the symbiont of *Macroplea mutica*. Picture of *M. mutica* kindly provided by Lech Borowiec. **g** Hive plot depicting perfect synteny across the symbiont genomes of five representative Donaciinae spanning the phylogenetic diversity of the subfamily. Coloring of genes in **f** and **g**: environmental information processing (green); genetic information processing (violet); metabolism (peach); RNA (yellow); cysteine and methionine metabolism (blue); phenylalanine, tyrosine, and tryptophan metabolism (pink); other amino acids metabolism (brown); other (gray).

We obtained full genome sequences for the symbionts of 26 reed beetle species from North America, Japan, and Europe, spanning the four genera *Plateumaris, Donacia, Macroplea*, and *Neohaemonia*. The genomes of all symbionts consisted of one chromosome of 453,917 to 516,927 bp (19.6–23.3% GC) and one plasmid of 4977–6762 bp (17.3–23.4% GC) (Table 1, Fig. 1a–f). The 403–454 protein-coding genes in both chromosome and plasmid showed perfect synteny across the symbiont strains (Fig. 1g, Supplementary Data 1), strongly indicating that genome erosion preceded the diversification of Donaciinae and resulted in a severely reduced and heavily AT biased genome in the ancestor of all extant reed beetles, which is surprising given the symbionts’ complex life cycle transitioning between extra- and intracellular localization. The primary metabolism of the symbionts retains pathways for glycolysis, oxidative phosphorylation, the biosynthesis of fatty acids, peptidoglycan, as well as most essential amino acids and the B-vitamin riboflavin, while pathways for other vitamins and non-essential amino acids as well as purines and pyrimidines are lacking. Interestingly, while the subunits of NADH-ubiquinone oxidoreductase and cytochrome d ubiquinol oxidase are retained in all of the symbionts, the ATP synthase complex is only present in symbionts of the host genus *Plateumaris*, but absent in *Donacia*, *Macroplea*, and *Neohaemonia*, suggesting that the symbionts of these taxa use the proton gradient generated by the respiratory complex for secondary transport reactions and meet their ATP demands by substrate-level phosphorylation alone or obtain additional ATP from the host.

**Table 1:** Characteristics of symbiont genomes across 26 Donaciinae host species in four genera.

	Chromosome		Plasmid	
Species	Size (bp)	GC content (%)	Size (bp)	GC content (%)
*Plateumaris braccata*	512,214	20.8	5287	21.5
*Plateumaris consimilis*	512,724	20.8	5136	20.4
*Plateumaris rustica*	498,937	20.1	5108	20.5
*Plateumaris pusilla*	514,699	20.3	5276	20.5
*Plateumaris sericea*	516,927	20.4	5142	19.1
*Neohaemonia nigricornis*	471,617	21.5	6337	20.1
*Macroplea appendiculata*	466,349	23.0	6432	22.5
*Macroplea mutica*	467,736	23.3	6327	22.7
*Donacia tomentosa*	484,935	22.1	6762	23.4
*Donacia versicolorea*	459,060	19.8	6568	19.7
*Donacia dentata*	458,942	19.9	6647	19.2
*Donacia semicuprea*	458,753	19.7	5734	17.3
*Donacia clavipes*	457,274	19.6	5754	18.0
*Donacia provostii*	453,917	20.0	6377	21.7
*Donacia cincticornis*	454,754	20.2	6683	21.0
*Donacia crassipes*	458,439	20.0	6529	20.7
*Donacia piscatrix*	459,119	20.0	6615	21.1
*Donacia proxima*	458,077	20.2	6453	21.0
*Donacia sparganii*	466,482	19.9	5238	19.5
*Donacia cinerea*	462,896	20.2	5119	19.8
*Donacia simplex*	461,708	20.0	5216	19.1
*Donacia vulgaris*	460,480	20.5	5262	20.7
*Donacia thalassina*	462,341	20.0	5482	20.0
*Donacia bicoloricornis*	458,627	20.6	5086	20.8
*Donacia marginata*	462,406	20.2	5198	18.9
*Donacia fulgens*	460,881	19.6	4977	18.5

### Symbionts provision essential amino acids and vitamins

**Fig. 2:**
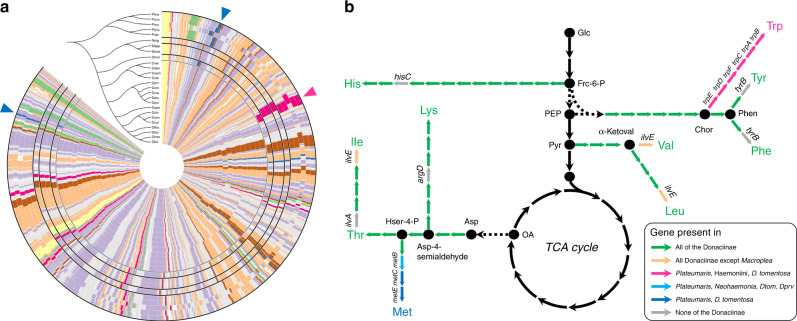
Evolution of (semi-)essential amino acid (AA) biosynthesis pathways in Donaciinae symbionts. **a** Comparison of symbiont genomes across 26 species of Donaciinae. Phylogenomic tree represents the relationships among symbionts, based on an alignment of 49 marker genes. Blue and magenta arrowheads indicate methionine and tryptophan biosynthesis genes, respectively, that have been lost in the symbionts of particular host taxa. Coloring of genes is the same as in Fig. 1f, g. **b** Schematic AA biosynthesis pathways as well as glycolysis and TCA cycle in reed beetle symbionts, with important intermediates and enzymes highlighted. Enzymatic steps in green are present across all symbiont genomes, those in gray are absent from all genomes. Colored steps indicate loss of enzymatic steps in particular taxa (see legend). Amino acids are colored according to the inferred capacity of the symbionts to produce them. Note that the loss of *ilvE* in *Macroplea* is assumed to be compensated for by alternative symbiont or host enzymes^22^.

Despite the severely reduced metabolism and the loss of all pathways for non-essential amino acids, the symbiont genomes of host species in the genus *Plateumaris* retain complete or almost complete pathways for the biosynthesis of the semi-essential amino acid tyrosine as well as all essential amino acids except arginine (Fig. 2). It is interesting to note that while most of the amino acid biosynthesis genes are located in the symbionts’ chromosome, a central enzyme of the aromatic amino acid biosynthesis pathway, i.e., a bifunctional chorismate mutase/prephenate dehydratase, is encoded on the plasmid in all species, providing an explanation for the selective constraint to maintain the plasmid throughout the evolution of the symbiosis. Of the five genes that were missing in otherwise complete amino acid biosynthesis pathways in the symbiont genomes, three were previously found in beetle genomes (*tyrB, ilvA,* and *ilvE*)^19–21^, and the enzymatic steps were hypothesized to be taken over by the host in the aphid-*Buchnera* symbiosis^22^. The remaining two genes (*hisC, argD*) encode aminotransferases, a group of enzymes that exhibits low substrate specificity^23^. Thus, other symbiont- or host-encoded aminotransferases likely fill in the gaps in histidine and lysine biosynthesis, as has been hypothesized for the amino acid supplementing symbiosis in mealybugs^24^. In conclusion, it seems likely that the *Plateumaris* symbionts—together with some beetle-encoded biosynthetic steps—are able to supplement their hosts’ nutrition with the semi-essential amino acid tyrosine as well as the essential amino acids histidine, methionine, tryptophan, phenylalanine, lysine, threonine, isoleucine, leucine, and valine, whereas they are unable to produce arginine or any of the non-essential amino acids.

While the biosynthetic pathways for the amino acids histidine, tyrosine, phenylalanine, lysine, threonine, isoleucine, leucine, and valine are retained in the symbionts of all four host genera, the symbionts of *Macroplea* and *Neohaemonia* lost the capacity to produce methionine, and all *Donacia* species except *D. tomentosa* lost the methionine and tryptophan biosynthesis pathways (Fig. 2). The presence of methionine biosynthesis genes in *D. tomentosa* strongly suggests two independent losses of methionine biosynthesis, one in the lineage leading to the Haemoniini (*Macroplea* + *Neohaemonia*), and one in the genus *Donacia* after the branching off of the ancestral *D. tomentosa* (Fig. 2). An additional loss of the gene *ilvE* encoding a branched-chain amino acid aminotransferase in the two *Macroplea* species’ symbionts is likely compensated by enzymes encoded in the host genome^22^, as the rest of the isoleucine, leucine, and valine biosynthesis pathways remains intact.

In addition to the symbionts’ capabilities to produce (semi-)essential amino acids, they retain a complete riboflavin biosynthesis pathway from GTP and ribulose-5-phosphate, except for the dephosphorylation step that has only recently been elucidated in *E. coli*^25^ and remains enigmatic in other bacterial symbionts^26^. As the symbionts lack the enzymatic machinery to produce purines, however, they likely rely on the host for the production of GTP, while they can synthesize ribulose-5-phosphate from glucose via the pentose phosphate pathway.

### Symbiont-encoded pectinases complement the hosts’ cellulases

**Fig. 3:**
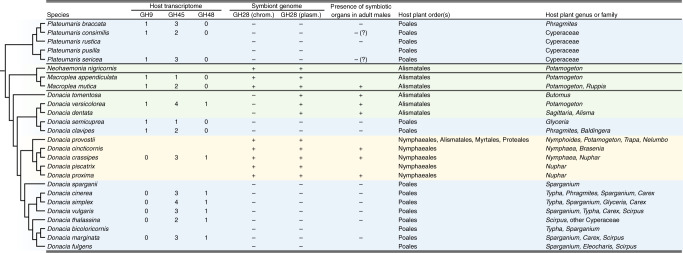
Plant cell wall degrading enzymes (PCWDEs) encoded by reed beetles and their symbionts. Numbers of PCDWE-encoding genes detected in the host gut transcriptomes and symbiont genomes, respectively, are indicated for Donaciinae species across four genera, and their congruence with the host plant preferences of the adult beetles and the presence of symbiotic bacteria in adult males are highlighted. The dendrogram on the left side depicts the phylogenetic relationships among the host species, reconstructed based on all 13 protein-coding genes in the mitochondrial genome. For the PCWDEs, the number of copies in the host transcriptome and the presence/absence of GH28 on the symbiont chromosome and plasmid is given, respectively. The presence of symbiotic bacteria in adult males was deduced from previous reports^15^ as well as the results of dissections and FISH in the present study. Host plant preferences are based on published reports^11,12,43,95,96^.

**Fig. 4:**
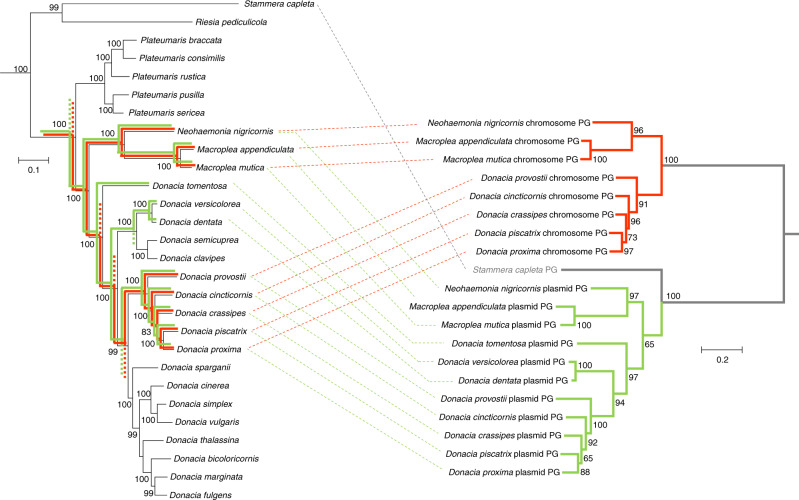
Phylogeny of Donaciinae symbiont pectinases perfectly mirrors that of the entire symbiont genomes. **a** Phylogenomic tree of Donaciinae symbionts based on 49 marker genes (FastTree analysis implemented in Kbase; local support values in percent are given at the nodes). Colored lines denote the reconstructed presences (solid lines) and losses (dashed lines) of chromosome-encoded (red lines) and plasmid-encoded polygalacturonases (green lines); **b** Phylogeny of pectinases encoded by the Donaciinae symbionts (FastTree analysis; local support values are given at the nodes). Branches leading to chromosome-encoded copies are highlighted in red, those leading to plasmid-encoded copies in green. The phylogenies of both pectinases are perfectly congruent with the phylogenomic tree of the symbionts.

In addition to the amino acid and riboflavin biosynthesis pathways that supplement the hosts’ nutrition, the genomes of some reed beetle symbionts encode a polygalacturonase (PG, i.e., GH28) in the chromosome and/or on the plasmid. Specifically, five of the 18 sequenced *Donacia* symbiont genomes as well as the two *Macroplea* and the *Neohaemonia* species contained both the chromosome-encoded and the plasmid-encoded PGs, while three additional *Donacia* species only retained the plasmid-encoded PG (Fig. 3). In accordance with the perfect synteny of the symbiont chromosomes and plasmids, the PGs were present in the same locations across symbiont genomes, strongly indicating shared ancestry. This is further corroborated by phylogenetic analysis, placing the chromosome-encoded and the plasmid-encoded copies of the different symbionts into two well-supported monophyletic clades, respectively (Supplementary Figs. 1–2). Furthermore, the relationships within these clades were perfectly congruent with a symbiont phylogeny reconstructed based on 49 chromosomal marker genes (Fig. 4). While the plasmid-encoded copies were most similar to the PG of the tortoise leaf beetle symbiont *Stammera capleta* within a clade of proteobacterial and acidobacterial PGs (Supplementary Fig. 1)^8^, the chromosome-localized PG was phylogenetically placed within a clade of bacterial PGs with mixed taxonomic affiliation (Supplementary Fig. 2).

**Fig. 5:**
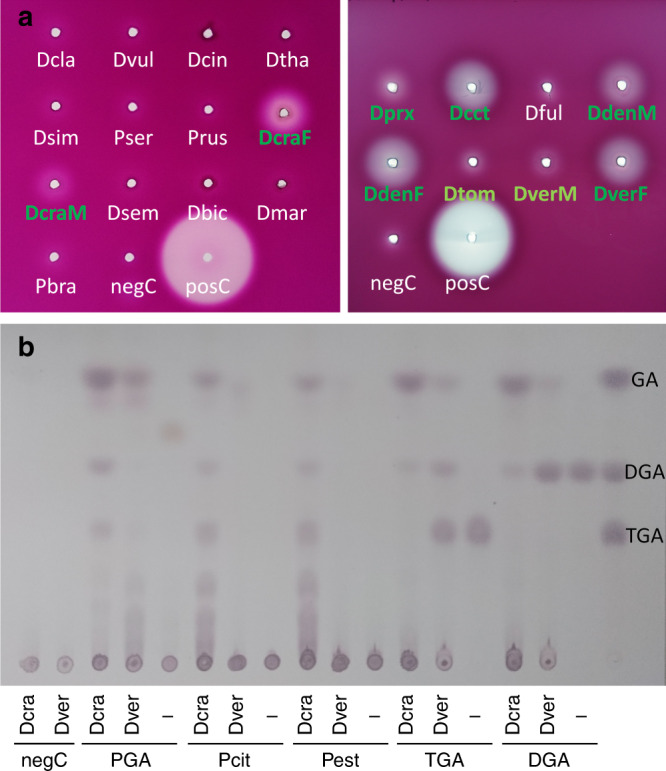
In vitro pectinase activity assays with gut extracts from different reed beetles. **a** Agar diffusion assays for polygalacturonase activity; gut extracts exhibiting clearly visible enzymatic effects are highlighted in dark green, those with weak activity in light green (Dcla *Donacia clavipes*, Dvul *Donacia vulgaris*, Dcin *Donacia cinerea*, Dtha *Donacia thalassina*, Dsim *Donacia simplex*, Pser *Plateumaris sericea*, Prus *Plateumaris rustica*, DcraF *Donacia crassipes* female, DcraM *Donacia crassipes* male, Dsem *Donacia semicuprea*, Dbic *Donacia bicolor*, Dmar *Donacia marginata*, Pbra *Plateumaris braccata*, Dprx *Donacia proxima*, Dcct *Donacia cincticornis*, Dful *Donacia fulgens*, DdenM *Donacia dentata* male, DdenF *Donacia dentata* female, Dtom *Donacia tomentosa*, DverM *Donacia versicolorea* male, DverF *Donacia versicolorea* female, negC negative control (no sample), posC positive control (*Phaedon cochleariae*)). Note that none of the Poales-feeding species showed polygalacturonase activity. **b** Thin-layer chromatography comparing the efficiency of *D. crassipes* (Dcra; symbiont genome encodes two pectinases) and *D. versicolorea* (Dver; symbiont genome encodes one pectinase) female gut extracts in breaking down different pectins (PGA polygalacturonic acid, deesterified (no methylation); Pcit pectin from citrus (60% of galacturonic acid residues are methylated); Pest pectin from citrus, esterified (85% of galacturonic acid residues are methylated); TGA trigalacturonic acid, DGA digalacturonic acid, GA galacturonic acid). Source data are provided as a Source Data file.

Sequencing of the gut transcriptomes of nine *Donacia*, two *Macroplea*, and three *Plateumaris* species yielded one to four copies of GH45s as well as one GH9 or GH48 per species, respectively (Fig. 3 and Supplementary Data 2–3). The presence of the latter two genes was almost perfectly complementary across the host transcriptomes, with only *D. versicolorea* encoding both a GH9 and a GH48. These results indicate the presence of host genome-encoded cellulases, but no pectinases. Thus, the pectinolytic activity of the symbionts complements the set of plant cell wall degrading enzymes (PCWDEs) in the host genome. Consistent with the predictions based on the host transcriptomes and symbiont genomes, in vitro activity assays with gut homogenates revealed cellulase activity in all species (Supplementary Fig. 3), while PG activity was confined to the species with pectinase-encoding symbionts (*D. crassipes, D. cincticornis, D. proxima, D. dentata*, and *D. versicolorea*) (Fig. 5a). The *D. tomentosa* gut homogenate exhibited only very weak PG activity despite the presence of a PG in the symbiont chromosome, but the assay was confined to a single specimen of this rare species whose gut appeared uncharacteristically thin during dissection, suggesting that the specimen may have been starving prior to collection.

To further characterize the activity of the symbiont-encoded PGs, thin-layer chromatography was performed with gut homogenates of *D. crassipes* and *D. cincticornis* (both PGs present), as well as *D. dentata* and *D. versicolorea* (only the plasmid-encoded PG present). The samples of all four species degraded polygalacturonic acid (PGA) (the homogalacturonan backbone of pectin) into the galacturonic acid monomers. However, the oligomers of galacturonic acid were more efficiently degraded by the species with both PGs present as opposed to only the plasmid-encoded PG (Fig. 3b and Supplementary Fig. 4). Thus, while the plasmid-encoded PG efficiently breaks down PGA, the chromosome-encoded PG appears to enhance the efficiency of galacturonan oligomer digestion.

In order to directly test the activity of the individual PGs, both the chromosome- and the plasmid-encoded PGs of the *M. mutica* (Mmut-cPG, Mmut-pPG) and the *D. crassipes* symbionts (Dcra-cPG, Dcra-pPG) were codon-optimized, synthesized, and transformed into *E. coli* for heterologous expression. Any attempt to express functional PGs failed except for Mmut-pPG, as Dcra-cPG solely formed inclusion bodies, and Mmut-cPG as well as Dcra-pPG were lost while elution fractions were buffer-exchanged (Supplementary Fig. 5a, b). To prevent protein loss, immunoprecipitation instead of buffer exchange was attempted, resulting in protein recovery on one hand but lack of activity on the other hand, even in the case of the otherwise functional Mmut-pPG. Enzymatic assays, however, demonstrated the pectolytic activity of the Mmut-pPG, which hydrolyzed PGA into dimers, trimers, and bigger galacturonic acid oligomers (Supplementary Fig. 5c). A weak activity towards break-down of galacturonic acid oligomers bigger than the trimer was also detected. Thus, although incompletely investigated due to the limited success with heterologous expression, these assays confirm that the plasmid-encoded symbiont PGs are active enzymes that contribute to pectin digestion in Donaciinae. Assuming that the homologous plasmid-encoded PGs possess a conserved function across reed beetles, the efficient degradation of small galacturonic acid oligomers by the gut homogenates of *D. crassipes* and *D. cincticornis* (both PGs present) cannot be explained with only the plasmid-encoded PG, based on the Mmut-pPG results. This further indicates a complementary role of the chromosome-encoded PG in galacturonic acid oligomer digestion, supporting our observations based on the in vitro activity of gut homogenates (Fig. 5b and Supplementary Fig. 4).

### Losses of pectinase genes correspond to host plant switches

**Fig. 6:**
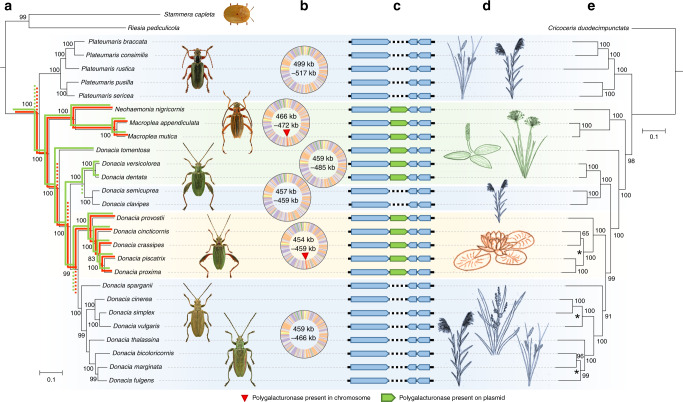
Phylogenomic analyses of Donaciinae and their symbiotic bacteria resolve the evolution of pectinolytic enzymes and their congruence with host plant preferences. **a** Phylogenomic tree of Donaciinae symbionts based on 49 marker genes (FastTree analysis implemented in Kbase; local support values in percent are given at the nodes). Colored lines denote the reconstructed presences (solid lines) and losses (dashed lines) of chromosome-encoded (red lines) and plasmid-encoded polygalacturonases (green lines). Next to the phylogeny, images of representative host taxa are given (from top to bottom: *Cassida rubiginosa* (outgroup), *Plateumaris braccata, Macroplea appendiculata, Donacia versicolorea, Donacia crassipes, Donacia cinerea, Donacia marginata*, pictures kindly provided by Lech Borowiec); **b** representative genomes of major symbiont clades, with range of genome sizes within the clade given inside each genome; a red triangle indicates the presence of a polygalacturonase (GH28) gene in the genome; **c** schematic alignment of plasmid sequences (linearized) across the 26 reed beetle symbionts; the presence of a polygalacturonase (GH28) gene on the plasmid is highlighted in green; **d** host plant preferences in the major Donaciinae clades, colored on the order level (blue: Poales; green: Alismatales; orange: Nymphaeales); background shading of Donaciinae clades corresponds to host plant preferences; **e** phylogenetic tree of reed beetles based on all 13 protein-coding genes in the mitochondrial genome; local support values in percent are given at the nodes; dashed lines connect the host taxa to their corresponding symbiont in **a**, and asterisks denote discrepancies between host and symbiont phylogenies.

Phylogenetic analyses of the beetle hosts based on all 13 protein-coding genes in the mitochondrial genome resulted in a well-supported phylogeny that recovered the four different genera as monophyletic clades, with the genus *Plateumaris* as sister group to all other Donaciinae, and the Haemoniini (*Macroplea* and *Neohaemonia*) as sister taxon to the genus *Donacia* (Fig. 6). Within *Donacia*, *D. tomentosa* was recovered as the earliest branching species, and the remaining *Donacia* species showed clear phylogenetic clustering according to their host plant preferences (Fig. 6d), as has been previously described^11^. In particular, seven clades of Donaciinae were recovered, three of which feed on plants in the order Poales (*Carex, Eleocharis, Typha, Sparganium, Phragmites*, and others), one feeds on Nymphaeales (mostly *Nymphaea* and *Nuphar*) and three on Alismatales (*Alisma, Sagittaria*, and *Potamogeton*).

Phylogenomic reconstructions of the symbionts based on 49 marker genes recovered a well-supported phylogeny that was almost perfectly congruent with the host phylogeny, with only three minor discrepancies occurring in the more recent splits within the Nymphaeales-feeding and Poales-feeding *Donacia* clades (Fig. 6a, e). Phylogenetic analyses of three plasmid-encoded genes (*trfA*, bifunctional chorismate mutase/prephenate dehydratase, and polygalacturonase) yielded perfect or near-perfect congruence with the phylogeny based on the set of symbiont chromosomal markers (Fig. 4 and Supplementary Figs. 6, 7), providing strong evidence for a shared ancestry of the plasmid and subsequent maintenance in all Donaciinae symbionts. The monophyletic clade of Donaciinae symbionts was most closely related to other bacterial symbionts of insects^27^, particularly the symbionts of human head lice (*Candidatus* Riesia pediculicola’)^28^ and *Cassida* leaf beetles (“*Candidatus* Stammera capleta”)^8^, as well as—more distantly—“*Candidatus* Buchnera aphidicola”, the primary symbiont of many aphids^29^.

Mapping the presence of the two PG genes onto the symbiont phylogeny revealed two clades containing both genes and two clades containing only the plasmid-encoded PG (Fig. 6a–c). Interestingly, the presence of PGs in the symbionts coincided perfectly with the beetles’ host plant preferences: Whereas the symbionts of all species feeding on Nymphaeales or Alismatales contained at least the plasmid-encoded PG, none of symbionts of the Poales-feeding species retained a PG (Fig. 6). The Nymphaeales-feeding *Donacia* clade and the completely aquatic Haemoniini (feeding on submerged leaves of Alismatales) additionally exhibited the genome-encoded copy, whereas the three *Donacia* species whose adults feed on erect or floating leaves of Alismatales (*Potamogeton natans, Sagittaria sagittifolia* and *Butomus umbellatus*, respectively) had lost the second PG. As the high degree of similarity and synteny across all symbiont genomes indicates that genome erosion likely predated the split of *Plateumaris* from the rest of the Donaciinae, horizontal acquisition of the PGs into the chromosome and plasmid, respectively, after the separation of *Plateumaris* seems unlikely. Thus, assuming the presence of the PGs in the ancestor of all Donaciinae symbionts, the phylogeny revealed four independent losses of the chromosome-encoded PG in lineages of Poales-feeding and Alismatales-feeding Donaciinae, and three independent losses of the plasmid-encoded PG, all in lineages of Poales-feeding taxa (Fig. 6).

### Non-pectinolytic symbionts are lost in adult males

**Fig. 7:**
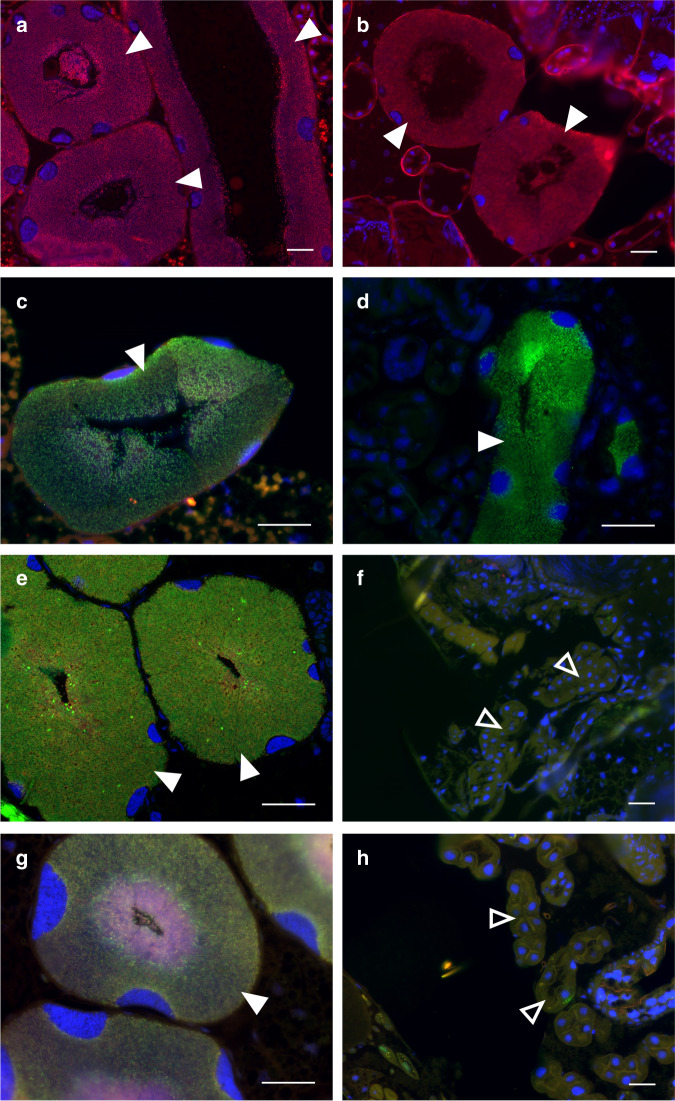
Fluorescence in situ hybridization micrographs of symbiotic organs in Donaciinae. Females (left panel) and males (right panel) of four representative species feeding on different host plants are shown (for fluorescence micrographs of 11 different species, see Supplementary Fig. 8). Note that different probes were used (see Supplementary Table 2), so the symbionts of different species are labeled in red (Cy3, **a**, **b**), green (Cy5, **c–e**), or yellow (Cy3 and Cy5, **g**). DAPI (blue) was used for general DNA counterstaining. Filled white arrowheads highlight symbiont-filled Malpighian tubules (symbiotic organs), empty arrowheads point to Malpighian tubules without symbionts. The following species are shown (host plant order given in brackets): **a**, **b** *Donacia crassipes* (Nymphaeales), **c**, **d** *Donacia dentata* (Alismatales), **e**, **f** *Donacia semicuprea* (Poales), **g**, **h** *Plateumaris sericea* (Poales). Note that only the Alismatales-feeding and Nymphaeales-feeding species show symbiont-bearing organs in adult males (**b**, **d**), whereas the males of Poales-feeding species are symbiont-free (**f**, **h**). By contrast, females carry symbionts in all species (**a**, **c**, **e**, **g**). Scale bars 50 µm.

Early descriptions of the Donaciinae symbiosis reported on differences in the presence of symbionts in adult males across species: while the males of seven Poales-feeding *Donacia* and one *Plateumaris* species were described to lack symbiotic organs entirely, enlargements of the Malpighian tubules were found for *Plateumaris sericea* and the Nymphaeales-/Alismatales-feeding *Donacia crassipes, D. tomentosa*, and *D. dentata*, albeit to a much lesser extent than in female beetles^15^. Based on this congruence of the apparent presence of male symbiotic organs with the beetles’ host plant preferences, we used fluorescence in situ hybridization to assess the presence and localization of symbionts in adult males and females across six *Donacia* and two *Plateumaris* species feeding on Poales (*D. cinerea, D. clavipes, D. semicuprea, D. simplex, D. thalassina, D. vulgaris, P. consimilis*, and *P. sericea*), two *Donacia* species feeding on Alismatales (*D. dentata* and *D. versicolorea*), and one on Nymphaeales (*D. crassipes*). The females of all species showed dense populations of symbionts in the epithelial cells (intracellularly) and the lumen (extracellularly) of the enlarged parts of the Malpighian tubules (Fig. 7 and Supplementary Fig. 8, left panels). By contrast, no symbionts could be discovered in the males of any of the Poales-feeding species, while the Alismatales-feeding and Nymphaeales-feeding males exhibited enlarged regions of the Malpighian tubules that were morphologically similar to those of the females, albeit much smaller, and contained dense populations of intracellularly and extracellularly localized symbionts (Fig. 7 and Supplementary Fig. 8, right panels). Hence, symbiont-containing organs in adult males appear to be restricted to species that feed on non-Poales host plants and harbor symbionts encoding pectinases in their chromosome and/or plasmid (Fig. 3). The previous description of the occasional presence of symbiotic organs in the Poales-feeding *P. sericea*^15^ could not be confirmed and warrants further investigation.

## Discussion

Donaciinae reed beetles engage in an unusual symbiotic association with vertically transmitted bacteria that transition between extracellular and intracellular localization over the course of the host beetle’s life cycle^15^. Specifically, the symbionts are localized extracellularly in the eggs’ jelly coat, intracellularly in the larval midgut caeca, and both intracellularly and extracellularly in the modified Malpighian tubules of old larvae and adults^15^. Despite being exposed to such different environments, the symbionts have experienced drastic genome erosion, resulting in genomes of 454–517 kbp in size and strongly AT-biased nucleotide composition (19.6–23.3% GC). Perfect synteny and strong conservation of shared metabolic pathways across the sequenced symbiont genomes spanning 26 host species in four genera indicates that genome erosion occurred rapidly after the origin of the symbiosis in the ancestor of all extant Donaciinae species, while afterwards few changes occurred during the 75–100 million years of Donaciinae diversification (age estimate based on Kölsch and Pedersen^11^). Such rapid genome erosion and long subsequent period of evolutionary stasis has previously been described for the obligate symbionts of aphids^30^ and subsequently many other insect symbioses^31^, suggesting that strong host-level selection for symbiont-conferred benefits prevents further genome erosion.

Reed beetle larvae exhibit an unusual ecology among beetles, feeding and developing attached to plant roots under water and completing their metamorphosis in a submerged cocoon^18^. Here we show that—despite severe genome erosion—the symbionts of all 26 beetle species we investigated encode a complete or almost complete set of biosynthetic pathways for the production of one semi-essential and nine essential amino acids as well as the B vitamin riboflavin to supplement the nutrition of the larvae. This observation supports early reports based on gut content analyses that the Donaciinae larvae feed on plant sap^14^, an unusual food resource for beetles. Relying on plant sap as the sole nutritional resource is widespread only within the order Hemiptera, and the sap-feeding taxa are invariably associated with intracellular symbionts provisioning essential amino acids and vitamins^32,33^. The convergent acquisition of symbionts in Hemiptera and Donaciinae for dietary supplementation hence supports the notion that sap-feeding insects generally require assistance by microbial mutualists to compensate for the nutrient-poor diet^32,34^. Concordantly, *M. appendiculata* larvae that were experimentally deprived of their bacterial symbionts by tetracycline treatment failed to construct their cocoons significantly more often than their symbiont-bearing counterparts^18^. Since early analyses suggested a proteinaceous nature of *Donacia* cocoons^35^, it is plausible that deprivation of amino acid-producing symbionts prevented the synthesis of cocoon proteins or impaired proper cocoon formation by causing general physiological problems due to amino acid starvation.

Mapping the amino acid biosynthesis pathways onto the symbiont phylogeny revealed that the Haemoniini and the *Donacia* species (except *D. tomentosa*) lost the capacity to produce methionine and/or tryptophan, and none of the Donaciinae symbionts can produce arginine. Interestingly, these three amino acids are among the four (including histidine) energetically most expensive essential amino acids, and it is exactly the pathways for these four most costly amino acids that are commonly outsourced from a primary endosymbiont to a subsequently acquired companion symbiont^36^. Hence, it has been speculated that the high energy expenditure may lead to the particularly rapid loss of these pathways in situations where the respective amino acids are available from alternative sources^36^. It remains unclear, however, how the Haemoniini and most *Donacia* species compensate for the loss of tryptophan and/or methionine biosynthesis genes in the symbiont genomes. A higher availability in the diet seems unlikely, as some *Donacia* species share the same Poales host plants with *Plateumaris* species that retained both biosynthetic pathways. Conceivably, however, the amino acid composition of the larval enzymes and the proteinaceous cocoon changed to accommodate the low availability of methionine and tryptophan from the diet alone, or an association with gut bacterial symbionts might provide an additional source of these amino acids.

The second function conferred upon some reed beetle hosts by their symbiotic bacteria appears to be the support in degrading plant cell walls through the production of PGs. Similarly, the extracellular symbiont ‘*Candidatus* Stammera capleta’ of the tortoise leaf beetle *Cassida rubiginosa* (Chrysomelidae, Cassidinae) contains a strongly reduced genome that is streamlined for the production of two pectinases, i.e. a PG and a rhamnogalacturonan lyase^8,37^. For *C. rubiginosa*, the pectinolytic symbiont was demonstrated to significantly benefit host fitness, reflected in a considerably higher likelihood to survive until adulthood of symbiotic vs. symbiont-depleted beetles^8^. As hypothesized for *Cassida*^8^, the acquisition of a symbiont encoding two pectinolytic enzymes by the ancestor of extant Donaciinae may have rendered the digestion of pectin more efficient and allowed for the secondary loss of the PG from the beetle’s own genome that had been acquired by horizontal gene transfer from an ascomycete fungus approximately 200 million years ago in the ancestor of the Phytophaga (i.e., Chrysomeloidea and Curculionoidea)^38,39^.

During the diversification of the Donaciinae, the chromosome-encoded and plasmid-encoded PGs were lost four and three times independently, respectively (Fig. 6). Interestingly, these losses perfectly coincide with host plant changes of the beetles, with all taxa switching their diet towards plants of the order Poales (sedges, reed, cattail, bur-reed, etc.) subsequently losing both PGs. As the primary cell walls of Poales are known to contain considerably lower amounts of pectin (1.3–5.7%) than those of other angiosperms (20–35%)^40–42^, selection to maintain the pectinases was likely relaxed, allowing for the loss of the corresponding genes. By contrast, the plasmid-encoded PG was maintained in all symbionts of Donaciinae species feeding on host plants of the orders Nymphaeales and Alismatales, facilitating the digestion of the pectin-rich cell walls by providing the capability to degrade PGA. Surprisingly, however, the genome-encoded PG was lost twice independently in specialist Alismatales-feeding lineages, i.e., *D. tomentosa* and *D. versicolorea* + *D. dentata*, respectively, likely reducing the host species’ ability to digest galacturonic acid oligomers that may occur due to incomplete breakdown of pectin by the plasmid-encoded PG. It remains enigmatic why the genome-encoded PG was lost twice in *Donacia* but maintained in the Haemoniini (*Macroplea* + *Neohaemonia*) that are reported to feed on similar host plants as *D. versicolorea*, i.e., species of the Alismatales genus *Potamogeton*. Conceivably, however, the completely aquatic lifestyle of the Haemoniini including the consumption of submerged leaves of *Potamogeton perfoliatus* and *P. pectinatus* by the adults^43^, rather than the floating leaves of *Potamogeton natans* as in *D. versicolorea*^11^, may confront them with different nutritional challenges.

As the pectinases were rapidly lost in all lineages feeding on Poales, the phylogenetic analysis allows for reconstructing the host plant usage of extinct Donaciinae taxa. Because (i) genome erosion likely preceded the diversification of the Donaciinae, given the perfect synteny and shared gene content in the chromosomes and plasmids, and (ii) horizontal acquisition of genes into the chromosome and plasmid of a genome-eroded symbiont seems very unlikely^31,44^, the two pectinases were probably present in the symbiont genome of the shared ancestor to all Donaciinae (Fig. 6). Under this scenario, the Donaciinae ancestor is inferred to have been feeding on plants with pectin-rich cell walls, possibly in the orders Alismatales (early branching monocots) or Nymphaeales (basal angiosperms), which is in contrast to the previously hypothesized Cyperaceae (Poales)-feeding lifestyle^45^. Our conclusion is further supported by the assumed closest relatives, the Criocerinae^46–48^, being originally monocotyledon feeders on families outside the pectin-poor Poales^49^. Interestingly, there were no reversals from Poales-feeding to other host plants (Fig. 6), so the loss of symbiont-encoded pectinases likely constrained the adaptive potential of the hosts and confined them to a diet that is poor in pectin. Thus, functional genomic analyses of symbiont-provided benefits can yield novel insights into their host insects’ ecology that would otherwise remain elusive. Additionally, the presence of pectinases in some Donaciinae symbiont genomes suggests that pectinolytic symbionts are more common in leaf beetles than previously thought, possibly occurring across the four subfamilies known to harbor symbiotic microbes, i.e., Cassidinae, Donaciinae, Eumolpinae, and Sagrinae^15,27,34,50^.

Microbial symbioses are often considered important sources of ecological innovations that provide opportunities for expanding dietary niche space^3,4^. Concordantly, symbiont-provided essential amino acids and pectinases were likely important for the exploitation of root sap and leaf material in larval and adult reed beetles, respectively, allowing for the shift to a semi-aquatic lifestyle. However, after switching host plants to pectin-poor Poales, the subsequent loss of symbiont-encoded pectinases in multiple beetle lineages apparently constrained dietary niche space, as no reversals to pectin-rich plants can be observed in the reed beetle phylogeny (Fig. 6)^11^. Hence, symbiotic microbes can initially expand their host’s niche space, but their genome erosion may subsequently constrain their host’s evolutionary potential and confine it to a particular ecological niche. While such symbiosis-inflicted constraints have been discussed in the context of the host’s viable temperature range^51^, they have rarely been considered for biotic niche dimensions, even though previous studies have shown that host plant specialization can be governed by microbial symbionts^52^, determining and limiting the host’s dietary niche space.

While immature and adult stages of hemimetabolous insects generally exploit the same food resources, the morphological and physiological changes during metamorphosis allow holometabolous insects to specialize on different diets in larvae and adults, a process known as adaptive decoupling^53^. Concordantly, benefits provided to holometabolous insects by microbial symbionts are often important only in a particular life stage, exemplified by the symbionts of grain weevils that support adult cuticle biosynthesis by provisioning aromatic amino acids, but are subsequently recycled by the host with the exception of a population used for transmission to the offspring in female beetles^54^. However, the Donaciinae symbionts are extraordinary in providing two different benefits to larvae and adults, respectively, supporting the nutritional needs of each of these life stages. While—akin to the sap-feeding Hemiptera—the larvae require supplementation of essential nutrients that are scarce in the plant sap-based diet, the folivorous adults benefit from digestive enzymes to break down the fastidious pectin in plant cell walls, resembling the situation in the tortoise leaf beetle *C. rubiginosa*^8^. The importance of pectinase-provisioning rather than the supply of amino acids to the adult Donaciinae beetles is supported by the observation that the symbionts are maintained in adult males only if they encode pectinases, whereas the females of all species contain well-defined organs, serving for the transmission of the symbionts to the offspring (Fig. 7 and Supplementary Fig. 8).

The provisioning of two different, life-stage specific benefits to Donaciinae beetles by their symbionts highlights the potential of holometabolous insects for adaptive decoupling of symbiont function and localization during metamorphosis^55^. Concordantly, the two different functionalities of the symbionts coincide with changes in localization during the host’s life cycle^15^. In the larva, the symbionts colonize the epithelial cells of specialized caeca associated with the anterior part of the midgut^15^. Although bacteria localized extracellularly in the digestive tract can also provision amino acids^56,57^, an intracellular localization may facilitate transfer of nutrients to the host. Concordantly, symbionts provisioning the full complement of essential amino acids and some vitamins in sap-feeding Hemiptera^32^ and omnivorous Carpenter ants^58^, individual amino acids in beetles^54,59^, or B vitamins in blood-feeding tsetse flies^60^ and bed bugs^61^, respectively, are likewise localized intracellularly. From the midgut organs, the symbionts are relocated into two enlarged Malpighian tubules in later larval stages, where they persist until adulthood in the females of all species as well as in the males of species with pectinase-encoding symbionts. Here, the symbionts occur both intracellularly and extracellularly, with the latter localization likely facilitating the transport of pectinases to the gut by obviating the need to cross the host cell membrane, as has been hypothesized for the pectinolytic symbionts of *C. rubiginosa*^8,37^. Although the localization of the symbionts in the Malpighian tubules that end at the midgut–hindgut junction may seem rather distal for the release of digestive enzymes that aid in breaking up plant cell wall components, a forward flow of digestive enzymes from the Malpighian tubules to the anterior midgut within the ectoperitrophic space has been reported previously in insects^62^, so it is conceivable that the pectinase exerts its activity in the midgut. The pectinolytic function may have initially selected for an extracellular route of transmission from the Malpighian tubules to the egg’s jelly coat, preventing an infection of the germline and the transition to a completely intracellular lifestyle. Thus, the complex journey of the Donaciinae symbionts may be the result of evolutionary constraints imposed by the dual functionality that required (and in the pectinolytic species still requires) a transition between intracellular and extracellular localization during the host’s life cycle.

Reed beetles are one of only a few beetle taxa that have transitioned from a terrestrial to an aquatic environment. Our results indicate that gamma-proteobacterial symbionts have been instrumental in allowing for this transition, by supporting the plant sap-based diet of the submerged larvae with limiting essential amino acids and riboflavin that are likely necessary for growth and cocoon formation. In addition, the reed beetle species feeding on pectin-rich plants are provided with polygalacturonases by their symbionts, complementing the hosts’ own set of cellulolytic enzymes, whereas the symbionts of species that secondarily switched to feeding on pectin-poor Poales lost the pectinases from their genomes. These findings change our view on the evolution of host plant usage in the Donaciinae and highlight the capacity of microbial symbionts to both expand and constrain ecological niche space in insects. Furthermore, the two different life stage-specific benefits conferred by the reed beetle symbionts provide an explanation for the complex relocalization events of the symbionts during the life cycle of their beetle host, yielding general insights into the functional constraints associated with intracellular and extracellular symbiotic lifestyles, respectively.

## Methods

### Beetle collection

Donaciinae species were collected as adult beetles in and around ponds and lakes in Germany, France, Japan, and the USA between 2005 and 2019 (Supplementary Table 1). Only the species *Macroplea appendiculata* was collected as larvae.

### DNA extraction

The symbiont containing organs of adult female beetles were dissected and stored at −80 °C. For the larvae of *M. appendiculata*, all internal organs were dissected for DNA extraction. DNA was extracted and purified with the Epicenter MasterPure^TM^ kit (Epicenter Biotechnologies, Madison, WI, USA).

### Diagnostic PCR

To confirm the presence of symbiont DNA in the samples, a specific primer pair for the symbionts of all European Donaciinae beetles was designed, targeting the 16S ribosomal DNA: Don_sym_F1 [5′- GAC TTR RAG GTT GTR AGC -3′] and Don_sym_R1 [5′- GAC TCY AAT CCG AAC TAM GAT A -3′]. The specificity of the primer pair was checked in silico using the Probe Match tool of the Ribosomal Database Project (RDP)^63^. For all samples, a diagnostic PCR with this primer pair was performed and the amount of DNA was measured using the Qubit® 2.0 Fluorometer (Thermo Fisher Scientific, Waltham, MA, USA) prior to genome sequencing.

### Sequencing of the *Macroplea mutica* symbiont reference genome

To obtain a high-quality genome of one symbiont, DNA extracted from the symbiotic organs of *Macroplea mutica* with the DNeasy Blood and Tissue kit (Qiagen, Hilden, Germany) was subjected to sequencing with PacBio (699 Mbp raw reads, average read length of 2260 bp; LGC Genomics, Berlin, Germany) and Illumina technologies (Illumina MiSeq, 250 bp mate-pair reads with 3 kbp library insert size, 30.8 million reads; GATC Biotech, Constance, Germany), respectively. The resulting PacBio reads were assembled into two circular symbiont contigs using Canu^64^, which were corrected with the Illumina reads by mapping in Geneious 11.0.5^65^.

### Genome sequencing of the symbionts of all other species

For each host species, DNA extracted from the symbiotic organs of one to four females beetles (or the larval internal organs for *M. appendiculata*) was used for symbiont genome sequencing. Sequencing was done commercially (StarSEQ GmbH, Mainz, Germany) with Illumina NextSeq 500 technology at a depth of approximately 5 million 150 bp paired-end reads per sample. The resulting raw reads were uploaded to the KBase Web server^66^, quality-checked with FastQC v1.0.4 (http://www.bioinformatics.babraham.ac.uk/projects/fastqc/), and adapter-trimmed and quality-trimmed using Trimmomatic v0.36^67^. Only read pairs for which both forward and reverse read passed the trimming were then assembled using SPAdes v3.12.0^68^. The resulting assembly was uploaded to Busybee^69^ to identify the contigs belonging to the symbiont genome. In Busybee, a taxonomic assignment of all fragments was carried out using Kraken^70^, to cluster contigs into different taxonomic groups and distinguish host and bacterial contigs. The data were further analyzed and visualized using RStudio version 1.1.453^71^ including the seqinr package^72^. All fragments with similar coverage, GC content around 20% and taxonomic annotation as Gammaproteobacteria were selected and blasted against the reference genome of the symbiont of *Macroplea mutica*. The matching fragments were saved in the correct order and orientation for further curation with Geneious 11.0.5^65^.

For most species, the SPAdes assembly and subsequent binning yielded a single circular contig for the plasmid, and one to three contigs covering the symbiont’s chromosome. For some species, all DNA fragments could be combined into one contig with de novo assembly of the SPAdes contigs using the assembler integrated in Geneious. In all other cases, a short sequence at the end of one fragment and at the beginning of the following fragment were aligned with the reference genome to merge both fragments correctly. In case that a part of the sequence was missing and a gap between both fragments occurred, these gaps were filled with the corresponding number of Ns. As the symbiont genomes were generally poor in repetitive sequences, the only major challenge was the assembly of the rRNA operons, as they were present in two copies within the genome, which could not be resolved with short-read sequencing. Hence, assembly of the rRNA operons was done by using the *M. mutica* symbiont genome as a reference, for which the long PacBio reads successfully resolved both copies. For all other genomes, all rRNA reads were incorrectly assembled into a single operon whose ends perfectly overlapped with both positions of the rRNA operons in the genome. To obtain closed draft genomes, the rRNA operon was therefore copied and automatically (Geneious) or manually assembled with the remaining contigs. As the *M. mutica* symbiont genome contains a tRNA (Glu-TTC) in one of the two otherwise identical rRNA operons, this tRNA may have been lost or duplicated in the process of assembling the other symbiont genomes. All resulting genomes were finally annotated with RAST v2.0^73^ as implemented in Kbase^66^.

### Symbiont genome analysis

The genes in the chromosomes of all symbionts were clustered into orthologous groups using OrthoMCL v.0.0.7^74^ (Supplementary Data 1). To determine the functional categories for each gene, BlastKOALA was used to assign KEGG Orthology (KO) identifiers based on the KEGG database^75–77^. Based on the hierarchical classification of the KO IDs, pathways were assigned to each gene. The annotated and categorized genes in the genomes were finally visualized with OmicCircos^78^. Synteny between different genomes was visualized with hive plots^79^, where the connections between genes was based on the OrthoMCL information of homologous proteins.

### Symbiont phylogenetic analyses

The sequences of 49 marker genes in the genomes of the Donaciinae symbionts, the tortoise leaf beetle symbiont *Stammera capleta*, and the 20 closest relatives in the Kbase database were aligned and used for phylogenomic analysis using FastTree 2^80^, as implemented in the “Insert Genomes into Species Tree” tool in Kbase^66^.

To assess the phylogenetic placement of the two pectinases, the translated coding sequences were extracted from the symbiont genomes and plasmids, respectively. Taking into account the low level of amino acid similarities between genome-encoded and plasmid-encoded GH28 proteins, we decided to perform two independent phylogenetic analyses. We used the same strategy for both analyses. We used either a genome-encoded or a plasmid-encoded GH28 protein sequence as a query for a BLASTP search of the ncbi_nr protein database and we recovered the first 250 hits. We eliminated redundancy at 90% identity level of both datasets using the CD-HIT Suite server^81^. Amino acid sequences were first aligned using MAFFT version 7^82^ and inspected visually in order to correct potential misaligned regions. Maximum likelihood-inferred phylogenetic analyses were performed on the IQ-TREE web server^83^, where the fittest evolutionary model was selected automatically. The robustness of the analysis was tested using 1000 bootstrap replicates.

### Extraction of mitochondrial genomes and phylogenetic analysis

For most beetle species, the contig containing the complete mitochondrial genome could easily be identified within the SPAdes assembly (see above) based on its size (between 14.3 and 15.9 kb), intermediate coverage (lower than the symbiont, but higher than host nuclear contigs) and low GC content (18.7–25.2%). For *D. cincticornis*, no contig containing the complete mitochondrial genome could be identified, so the raw reads were mapped against the mitochondrial genomes of all 25 other Donaciinae species, yielding 35 contigs that captured approximately 56.3% of the mitochondrial genome. The partial (*D. cincticornis*) or complete (all other species) nucleotide sequences of all 13 protein-coding genes in the beetles’ mitochondrial genomes were extracted, aligned, concatenated, and used for a phylogenetic analysis using *Cricoceris duodecimpunctata* (NC 003372) as an outgroup. Phylogenetic trees were reconstructed using FastTree (GTR model)^80^, PhyML (GTR model, 100 bootstrap replicates)^84^, and RAxML (GTR + Gamma model, 100 bootstrap replicates, dataset partitioned by gene and codon position (first and second positions combined, third positions separated)^85^, respectively. As all three methods yielded identical tree topologies, only the FastTree phylogeny is displayed.

### Fluorescence in situ hybridization of symbiotic organs

In order to assess the presence and localization of symbiotic bacteria in adult males and females across species, fluorescence in situ hybridization (FISH) was performed on one or two male and female specimens, respectively, of eight *Donacia* and *Plateumaris* species feeding on Poales (*D. cinerea, D. clavipes, D. semicuprea, D. simplex, D. thalassina, D. vulgaris, P. consimilis*, and *P. sericea*), two *Donacia* species feeding on Alismatales (*D. dentata* and *D. versicolorea*), and one on Nymphaeales (*D. crassipes*). Elytra, head, and legs were removed from the specimens prior to fixation in either Carnoy’s fixative (ethanol, chloroform, acetic acid in a ratio of 6:3:1) or 4% formaldehyde (FA) in PBS. After up to 4 h of fixation, FA-fixed samples were washed in water and then dehydrated in an increasing butanol series, whereas Carnoy-fixated specimens were washed in butanol. Subsequently, specimens were embedded in Technovit®8100 (Kulzer GmbH, Wehrheim, Germany) according to the manufacturer’s protocol, and then subjected to semi-thin sectioning (8 µm) on a Leica RM-2245 rotary microtome (Leica, Wetzlar, Germany). Finally, sections were subjected to FISH as described previously^86^, using a combination of two of the three fluorescent oligonucleotide probes Don-Sym (specific to Donaciinae symbionts, 5′-GCTYACAACCTYYAAGTC-3′), EUB338 (general for Eubacteria, 5′-GCTGCCTCCCGTAGGAGT-3′)^87^, and EUB784 (general for Eubacteria, 5′-TGGACTACCAGGGTATCTAATCC-3′)^88^, labeled with Cy3 or Cy5, respectively, as well as DAPI as a general DNA counterstain (Supplementary Table 2). Briefly, samples were hybridized for 90 min at 60 °C in hybridization buffer (0.9 M NaCl, 0.02 M Tris/HCl pH 8.0, 0.01% SDS) containing 5 µl of each probe and 5 µg ml^−1^ DAPI. Two wash steps with pre-warmed washing buffer (0.1 M NaCl, 0.02 M Tris/HCl pH8.0, 0.01% SDS, 5 mM EDTA), the second for 20 min at 60 °C, as well as rinsing with dH_2_O served to remove residual probe. After drying at room temperature, slides were covered with VectaShield® (Vector Laboratories Ltd., Peterborough, UK) and inspected on an AxioImager.Z2 fluorescence microscope (Zeiss, Jena, Germany).

### Host transcriptome sequencing and identification of PCWDEs

To assess the beetle hosts’ genetic repertoire for PCWDEs, live beetles were briefly cooled down at −20 °C to immobilize them, and then their digestive tract was dissected, flash-frozen in liquid nitrogen and stored at −80 °C. Total RNA was extracted from the beetles’ midguts using the innuPrep DNA/RNA Mini kit (Analytik Jena, Jena, Germany) following the manufacturer’s instructions. A DNase treatment was then performed to remove potential genomic DNA contaminations using TURBO™ DNase (Invitrogen, Carlsbad, CA, USA) for 30 min at 37 °C. Purification and concentration of the RNA samples were achieved using the RNeasy MinElute Clean up Kit (Qiagen, Hilden, Germany) following the manufacturer’s protocol. Control of the quality of the RNA samples was determined using the RNA 6000 Nano LabChip kit on an Agilent 2100 Bioanalyzer (both Agilent Technologies, Santa Clara, CA, USA) according to the manufacturer’s instructions.

RNA-Seq was performed at the Max Planck Genome Center (Cologne, Germany) where poly(A)+-RNA was first enriched before being fragmented to an average of 300–350 nucleotides. Then, a TruSeq compatible, directional library was prepared for each sample using dual-indexed adapter tags. Sequencing was carried out on a HiSeq3000 sequencing platform (Illumina, CA, USA) using paired-end (2 × 150 bp) reads. Quality control measures, including the filtering of high-quality reads based on fastq file scores, the removal of reads containing primer/adapter sequences and trimming of the read length, were carried out using CLC Genomics Workbench v11.0 (Qiagen, Hilden, Germany). Several assemblies were performed for each sequencing dataset. These assemblies differed by the number of randomly selected read pairs that were included (Supplementary Data 2). The quality of each assembly was assessed by performing a BUSCO (Benchmarking Universal Single-Copy Ortholog)^89^ analysis on an in-house Galaxy server.

All RNA samples were processed in the same way for RNA-Seq, except the one for *Macroplea mutica*. In this case, the TruSeq compatible, directional library was prepared using single-indexed adapter tags and was multiplexed with other beetle-derived libraries on the same sequencing lane. After assembly of the resulting sequencing dataset, we realized that the *M. mutica* assembly was cross-contaminated with sequences from other datasets which were sequenced on the same lane which made subsequent analyses difficult. We used the protocol described by Peters et al.^90^ in order to cure these RNA-Seq data from cross-contamination. We performed cross-BLAST searches, using BLASTN, between the *M. mutica* transcriptome assembly and all other assemblies corresponding to samples sequenced in the same run. Transcripts that shared nucleotide sequence identity of at least 98% over a length of at least 180 bp between two or more assemblies were identified. If the relative coverage of two transcripts originating from two different assemblies differed >2-fold, the transcript with the lower relative coverage was assumed to be a contaminant and was removed from the corresponding assembly.

Transcriptome assemblies of reed beetle species were then screened for the presence of transcripts encoding putative plant cell wall degrading enzymes (PCWDEs) using TBLASTN and previously characterized beetle-derived PCWDE sequences^91,92^. In parallel, the transcriptome with the highest number of complete single-copy orthologous genes, according to the BUSCO analysis, per species was screened for its complement of carbohydrate-active enzymes (CAZyme) using the dbCAN2 meta server (http://bcb.unl.edu/dbCAN2/index.php)^93^ (Supplementary Data 3).

### In vitro analysis of plant cell wall degrading capabilities

Freshly dissected and frozen guts (−80 °C, see above) were used for enzymatic assays to characterize the ability of the beetle and its symbionts to digest plant cell wall components. Guts were thawed on ice, pooled for each species and subjected to homogenization in a precooled Tissue LyserLT (Qiagen, Hilden, Germany) using 25 µl of homogenization buffer for each gut and three metal beads per tube. Subsequently, 50 mM citrate/phosphate buffer pH 5.0 including protease inhibitor cocktail (complete EDTA-free, Roche, Basel, Switzerland) were added to the pooled guts and samples were shaken for 1 min at 50 Hz. Homogenates were centrifuged at 16,000×*g* at 4 °C for 2 min to pellet remnants of gut tissue. Supernatants were directly used for agarose diffusion assays in 1% agarose Petri dishes containing either 0.1% demethylated PGA from citrus (PGA; degree of methylation DM 0%) (Megazyme, Wicklow, Ireland) or 0.1% carboxymethylcellulose (CMC; Sigma, St. Lois, MO, USA) and 50 mM citrate/phosphate buffer pH 5.0^94^. Two-millimeter holes were made into the agarose, and 10 μl of supernatant from gut homogenates were added to each hole. Agarose plates were incubated for 16 h at 40 °C. Activity was revealed after 1 h of incubation with 0.1% Congo Red solution (for CMC) or 2 h with 0.1% Ruthenium red solution (for PGA) at room temperature; each plate was then destained with 1 M NaCl or distilled water (for ruthenium red) until pale activity zones appeared against a dark red background. For qualitative analysis of breakdown products of the gut homogenates by thin layer chromatography (TLC), supernatants were first subjected to desalting with Zeba Desalt Spin Columns with a 7 kDa cutoff (Thermo Fisher Scientific, Waltham, MA, USA) according to the manufacturer’s guidelines to remove impurities of low molecular weight. Desalted samples were then used for TLC 20 µl enzyme assays set up as follows: 14 µl of each sample was incubated with either 0.4% pectin polymer or 10 µg of pectic oligomer of galacturonic acid (GalA) in a 20 mM citrate/phosphate buffer pH 5.0 at 40 °C for about 16 h^38^. Enzymatic activity was tested on the following pectin polymers: demethylated PGA from citrus (same as above), pectin from citrus peel (DM 60%) and esterified pectin from citrus fruit (DM 85%), both from Sigma (St. Louis, MO, USA). In addition, the following pectic oligomers of galacturonic acid (GalA) were tested: GalA heptamer/octamer mixture and tetramer (both from Elicityl, Crolles, France), trimer and dimer (both from Santa Cruz Biotechnology, TX, USA). The whole assay volumes were used for TLC afterwards. Samples were applied to TLC plates (Silica gel 60, 20 ×20 cm, Merck, Kenilworth, NJ, USA) in 2.5 µl steps and plates were developed ascending with ethyl acetate: glacial acetic acid: formic acid: water (9:3:1:4) for about 120 min. After drying, carbohydrates were stained by spraying plates in 0.2% (w/v) orcinol in methanol: sulfuric acid (9:1), followed by a short heating until spots appeared. The reference standard contained 2 µg each of GalA, GalA dimer, and GalA trimer.

### Heterologous expression of symbiont-encoded pectinase genes

Glycoside hydrolase family 28-coding genes of *D. crassipes* and *M. mutica* symbionts were synthesized by the company Genscript (Piscataway, NJ, USA), including codon optimization for expression in *E*. *coli*, and were subsequently cloned into pET-22b(+) in frame with a C-terminal V5 epitope and a 6×His tag. Both, plasmid-located (Dcra-pPG, Mmut-pPG) and chromosome-located (Dcra-cPG, Mmut-cPG) GH28 genes were synthesized (see Supplementary Data 4 for codon optimized sequences). Heterologous expression was performed using the Overnight Express Autoinduction System 1 by Novagen according to the manufacturers protocol (Merck, Kenilworth, NJ, USA) with slight modifications as follows. Autoinduction cultures were directly inoculated with single colonies picked from plated BL21 Star (DE3) transformations and were incubated in baffled flasks (50 ml medium/250 ml flask) at 18 °C and 200 rpm for 40 h. Cells were pelleted and subsequently lysed with Novagen BugBuster 10× (Merck, Kenilworth, NJ, USA) in Immobilized Metal Affinity Chromatography (IMAC) Binding buffer (see below) supplemented with Lysonase by rotating the samples at room temperature for 30 min. Samples were centrifuged and the supernatant was subjected to IMAC purification on a column self-packed with 1 ml HisPur cobalt resin. After applying samples in IMAC Binding buffer (50 mM sodium phosphate buffer pH 7.7, 0.5 M sodium chloride, protease inhibitor) on the pre-equilibrated column, the resin was washed extensively (50 mM sodium phosphate buffer pH 7.7, 0.3 M sodium chloride, 10 mM imidazole, protease inhibitor) and eluted three times (sodium phosphate buffer pH 7.4, 0.3 M imidazole, protease inhibitor) with 1 min incubation time for each elution step. Elution fractions e0 were subjected to buffer exchange against 50 mM citrate/phosphate buffer pH 5.0 on Zeba Spin Desalting columns with a 7 kDa cutoff (Thermo Fisher Scientific, Waltham, MA, USA). Alternatively, recombinant proteins from elution fractions were pulled down by immunoprecipitation using anti-V5 agarose beads (Bethyl Laboratories, Montgomery, TX, USA) as follows. The complete 500 µl of the elution e1 were mixed with 20 µl of the agarose bead slurry and incubated rotating over night at 4 °C. The mixture was centrifuged at 1000×*g* at 4 °C for 2 min to pull down the beads. Beads were washed three times with 500 µl of 50 mM citrate/phosphate buffer pH 5.0 and subsequently re-suspended in 100 µl of water. Success of expression and purification was monitored by Western Blot using a horseradish peroxidase (HRP) coupled V5 tag monoclonal antibody (dilution 1:10,000) and the SuperSignal West Extended Duration Substrate (both Thermo Fisher Scientific, Waltham, MA, USA). Both, buffer exchanged elution fractions as well as immuno-precipitated and re-suspended recombinant GH28 proteins were used for enzymatic assays (TLC).

### Statistics and reproducibility

Symbiotic organs were dissected from 2 to 10 specimens per host species, and localization was consistent throughout, as represented in Fig. 1d. Fluorescence in situ hybridization to localize the microbial symbionts in adult beetles’ Malpighian tubules (Figs. 1e, 7, and Supplementary Fig. 8) was performed on one (*Donacia cinerea*; *Donacia clavipes; Donacia crassipes; Donacia dentata; Donacia semicuprea; Donacia simplex; Donacia thalassina; Donacia vulgaris* male*; Plateumaris sericea*) or two (*Donacia versicolorea; Donacia vulgaris* females*; Plateumaris consimilis*) specimens per species and sex, yielding consistent results. The heterologous expression of GH28 proteins (Supplementary Fig. 5) was performed three times. The success of heterologous expression and subsequent IMAC was monitored three times but the pull down using anti-V5 agarose beads was just performed and monitored once. Replicated experiments yielded consistent results.

### Reporting summary

Further information on research design is available in the Nature Research Reporting Summary linked to this article.

## Supplementary information

Supplementary Information

Peer Review File

Description of Additional Supplementary Files

Supplementary Data 1

Supplementary Data 2

Supplementary Data 3

Supplementary Data 4

Reporting Summary

## Data Availability

Symbiont genome and host mitochondrial genome sequences are available in the SRA of NCBI under BioProject number PRJNA587602, and host transcriptome sequencing data under PRJNA575113. The following databases were used in this study: KBase, NCBI protein database, KEGG database, CAZy database, dbCAN HMM database and Hotpep peptide database. Source data are provided with this paper.

## Source data

Source Data
