# Supplementary material for: Bacterial symbionts support larval sap feeding and adult folivory in (semi-)aquatic reed beetles
Source: Nat Commun. 2020 Jun 11;11:2964. doi: 10.1038/s41467-020-16687-7 (PMC7289800; doi:10.1038/s41467-020-16687-7)
Supplement: Supplementary file 1 — Supplementary Information [file 41467_2020_16687_MOESM1_ESM.pdf]

Supplementary Information

Bacterial symbionts support larval sap feeding and adult folivory in aquatic reed beetles

Frank Reis[1,2, §], Roy Kirsch[3, §], Yannick Pauchet[3, §], Eugen Bauer[1], Lisa Carolin Bilz[1], Kayoko Fukumori[4], Takema Fukatsu[4], Gregor Kölsch[5,6], Martin Kaltenpoth[1,*]

[1]Evolutionary Ecology, Institute for Organismic and Molecular Evolution (iomE), Johannes Gutenberg University, Hanns-Dieter-Hüsch-Weg 15, 55128 Mainz, Germany

[2]Present address: Plant Evolutionary Ecology, Institute of Evolution and Ecology, University of Tübingen, Auf der Morgenstelle 5, 72076 Tübingen, Germany.

[3]Department of Entomology, Max Planck Institute for Chemical Ecology, Hans-Knöll-Str. 8, 07745 Jena

[4]Bioproduction Research Institute, National Institute of Advanced Industrial Science and Technology, Tsukuba 305-8566, Japan

[5]Molekulare Evolutionsbiologie, Institut für Zoologie, Universität Hamburg, Martin-Luther-King-Platz 3, 20146 Hamburg, Germany.

[6]Present address: Maasen 6, 24107 Kiel, Germany.

[§]contributed equally

*corresponding author, mkaltenpoth@uni-mainz.de

Supplementary Tables

Supplementary Table 1: Collection sites of Donaciinae species used in this study, and the host plants from which they were collected (if known).

Species	Location	Collected from host plant
Donacia bicoloricornis	Sanmu city, Chiba, Japan	unknown
Donacia cincticornis	Michigan, USA	unknown
Donacia cinerea	Hettenleidelheim, Germany	*Typha angustifolia*
Donacia clavipes	Hettenleidelheim, Germany	*Phragmites australis*
Donacia crassipes	Darmstadt, Germany	*Nymphaea spec.*
Donacia dentata	Haaler Au, Rendsburg, Schleswig-Holstein, Germany	*Sagittaria sagittifolia*
Donacia fulgens	Marlboro, Vermont, USA	*Sparganium spec.*
Donacia marginata	Horbach, Germany	*Sparganium spec.*
Donacia piscatrix	Ontario, Canada	unknown
Donacia provostii	Tsukuba city, Ibaraki, Japan	unknown
Donacia proxima	Whitingham, Vermont, USA	unknown
Donacia semicuprea	Niederhausen, Germany	*Glyceria spec.*
Donacia simplex	Mainz-Gonsenheim, Germany	*Glyceria spec.*
Donacia sparganii	Haaler Au, Rendsburg, Schleswig-Holstein, Germany	*Sparganium emersum*
Donacia thalassina	Hettenleidelheim, Germany	*Carex spec.*
Donacia tomentosa	Brécy-Brières, France	*Butomus umbellatus*
Donacia versicolorea	Spechtshausen, Germany	*Potamogeton natans*
Donacia vulgaris	Mainz-Bretzenheim, Germany	*Typha latifolia*
Macroplea appendiculata	Kreis Plön, Schleswig-Holstein, Germany	*Myriophyllum spicatum*
Macroplea mutica	Orth, Fehmarn, Germany	*Potamogeton pectinatus*
Neohaemonia nigricornis	Ontario, Canada	unknown
Plateumaris braccata	Bad Kreuznach, Germany	*Phragmites spec.*
Plateumaris consimilis	Darmstadt, Germany	*Carex spec.*
Plateumaris rustica	Sippersfeld, Germany	*Carex spec.*
Plateumaris sericea	Niedersteinbach, Germany	*Carex spec.*
Plateumaris pusilla	Lost Lake, Santiam Junction, Oregon, USA	*Carex spec.*

Supplementary Table 2: Oligonucleotide probes for localization of symbiotic bacteria in female and male reed beetles by fluorescence in situ hybridization.

Species	Cy3 (red)	Cy5 (green)
Donacia cincticornis	EUB748	EUB338
Donacia cinerea	EUB338	Don_Sym
Donacia clavipes	EUB748	EUB338
Donacia crassipes	EUB338	Don_Sym
Donacia dentata	EUB748	EUB338
Donacia semicuprea	EUB338	Don_Sym
Donacia simplex	EUB748	EUB338
Donacia thalassina	EUB338	Don_Sym
Donacia versicolorea	EUB748	EUB338
Donacia vulgaris	EUB748	EUB338
Plateumaris consimilis	EUB338	Don_Sym
Plateumaris sericea	EUB338	Don_Sym

Supplementary Figures

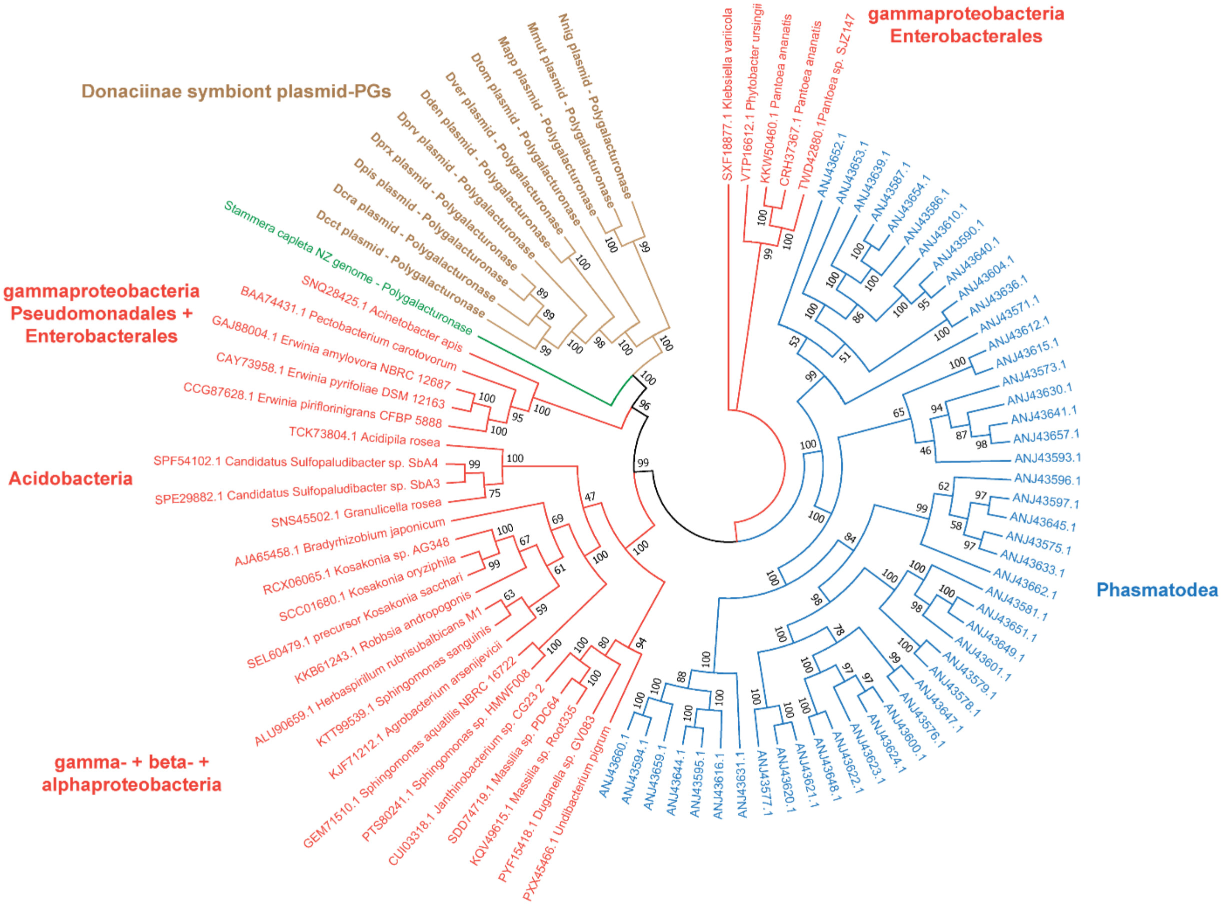

Supplementary Figure 1: Phylogenetic relationships of the plasmid-encoded GH28s of Donaciinae symbionts and their counterparts from other bacteria and insects. A maximum likelihood analysis was performed with 1000 bootstrap replicates. Bootstrap values are indicated next to the branches. The best-fit substitution model for this dataset was determined in IQ-TREE to be the “Whelan and Goldman” (WAG) model, incorporating a FreeRate model (five categories) of evolutionary rate differences among sites (+R). Taxonomic groups are differentially colored. Donaciinae symbiont plasmid-derived GH28s: brown; *Stammera capleta* genome-encoded GH28: green; other bacteria: red; Phasmatodea-derived sequences: blue. Accession numbers of the sequences used are indicated.

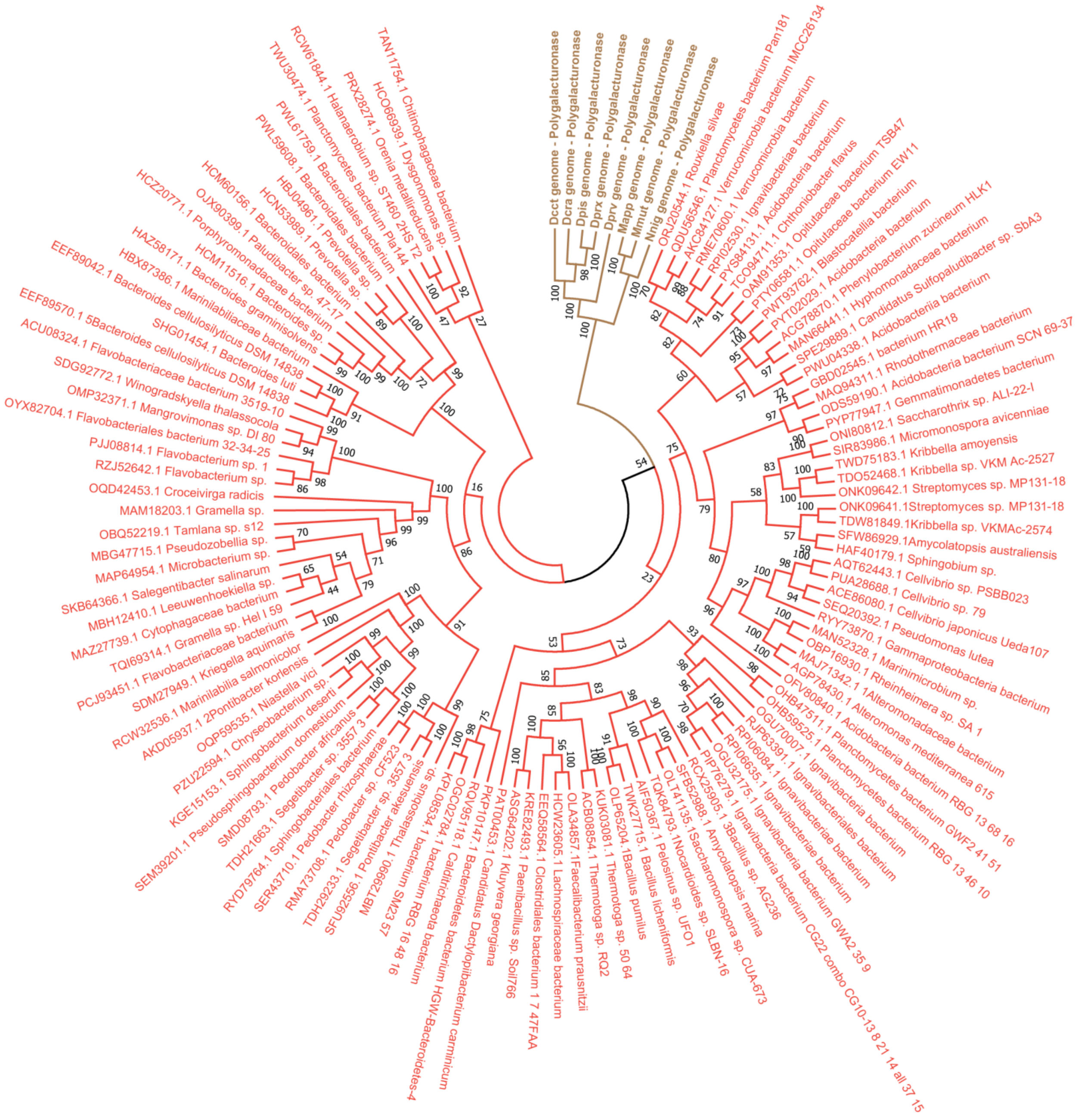

Supplementary Figure 2: Phylogenetic relationships of the chromosome-encoded GH28s of Donaciinae symbionts and their counterparts from other bacteria. A maximum likelihood analysis was performed with 1000 bootstrap replicates. Bootstrap values are indicated next to the branches. The best-fit substitution model for this dataset was determined in IQ-TREE to be the “Le and Gascuel” (LG) model, incorporating a FreeRate model (seven categories) of evolutionary rate differences among sites (+R). Taxonomic groups are differentially colored. Donaciinae symbiont genome-derived GH28s: brown; other bacteria: red. Accession numbers of the sequences used are indicated.

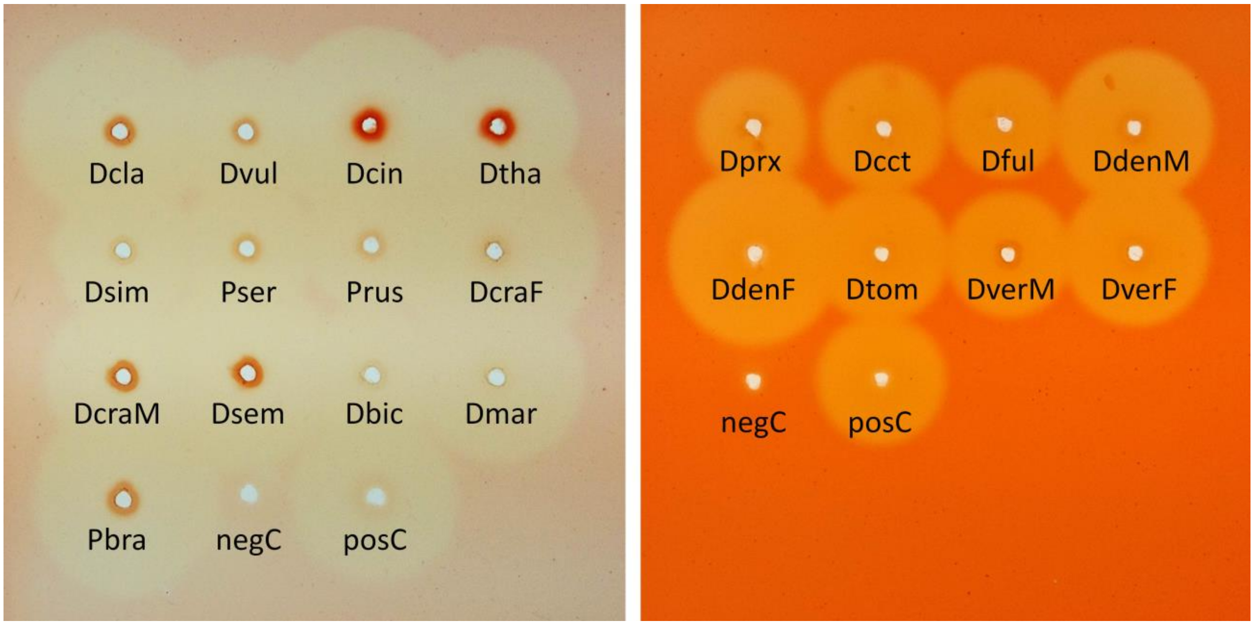

Supplementary Figure 3: *In vitro* cellulase activity assays with gut extracts from different reed beetles, demonstrating that all beetles contain cellulases in their gut (Dcla: *Donacia clavipes*, Dvul: *Donacia vulgaris*, Dcin: *Donacia cinerea*, Dtha: *Donacia thalassina*, Dsim: *Donacia simplex*, Pser: *Plateumaris sericea*, Prus: *Plateumaris rustica*, DcraF: *Donacia crassipes* female, DcraM: *Donacia crassipes* male, Dsem: *Donacia semicuprea*, Dbic: *Donacia bicolor*, Dmar: *Donacia marginata*, Pbra: *Plateumaris braccata*, Dprx: *Donacia proxima*, Dcct: *Donacia cincticornis*, Dful: *Donacia fulgens*, DdenM: *Donacia dentata* male, DdenF: *Donacia dentata* female, Dtom: *Donacia tomentosa*, DverM: *Donacia versicolorea* male, DverF: *Donacia versicolorea* female, negC: negative control (no sample), posC: positive control (*Phaedon cochleariae*)). Source data are provided as a Source Data file.

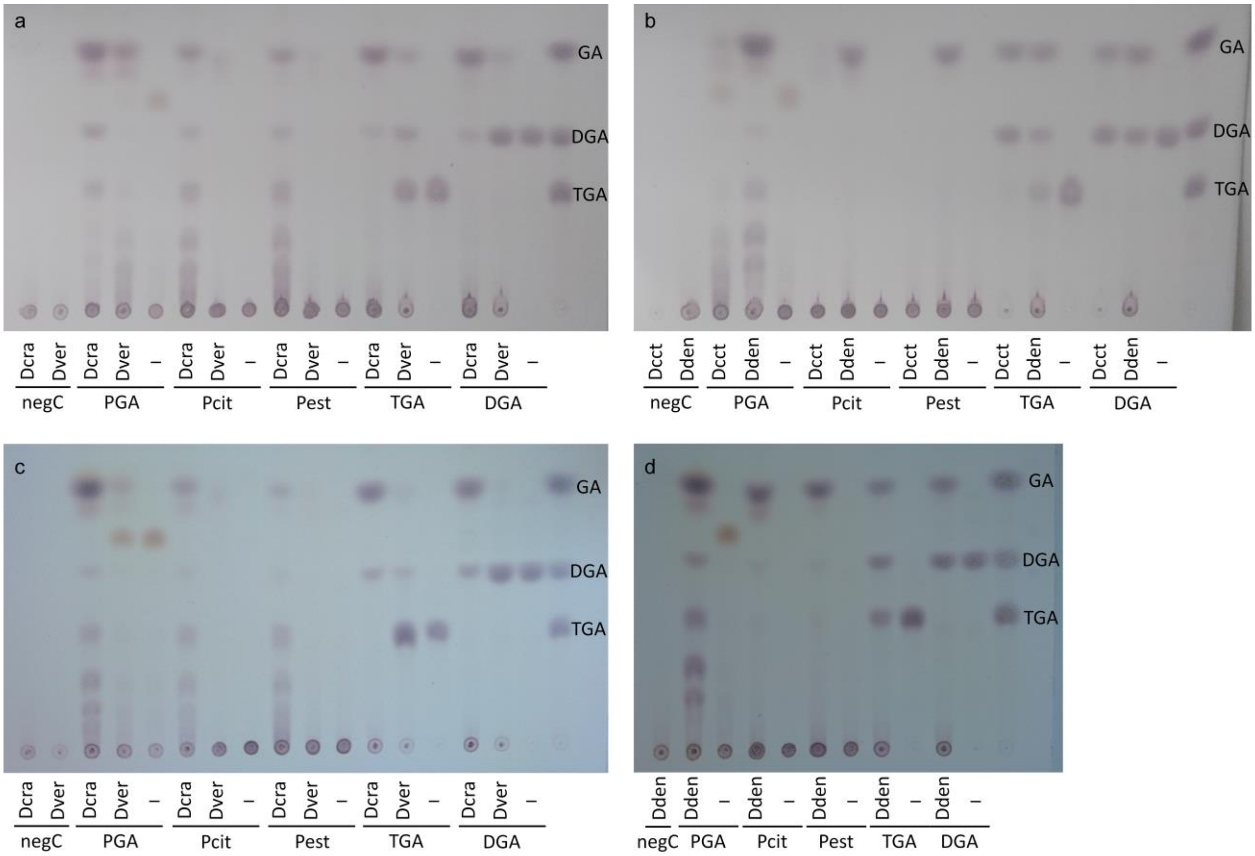

Supplementary Figure 4: Thin-layer chromatography assessing the efficiency of gut extracts from reed beetle species harboring symbionts with one or two pectinases, respectively, in breaking down different pectins. (a-b) Females and (c-d) males. Dcra: *D. crassipes* (symbiont genome encodes two pectinases); Dver: *D. versicolorea* (symbiont genome encodes one pectinase); Dcct: *D. cincticornis* (symbiont genome encodes two pectinases); Dden: *D. dentata* (symbiont genome encodes one pectinase). Substrates: PGA: polygalacturonic acid, deesterified (no methylation); Pcit: pectin from citrus (60 % of galacturonic acid residues are methylated); Pest: pectin from citrus, esterified (85 % of galacturonic acid residues are methylated); TGA: trigalacturonic acid; DGA: digalacturonic acid; GA: galacturonic acid. Source data are provided as a Source Data file.

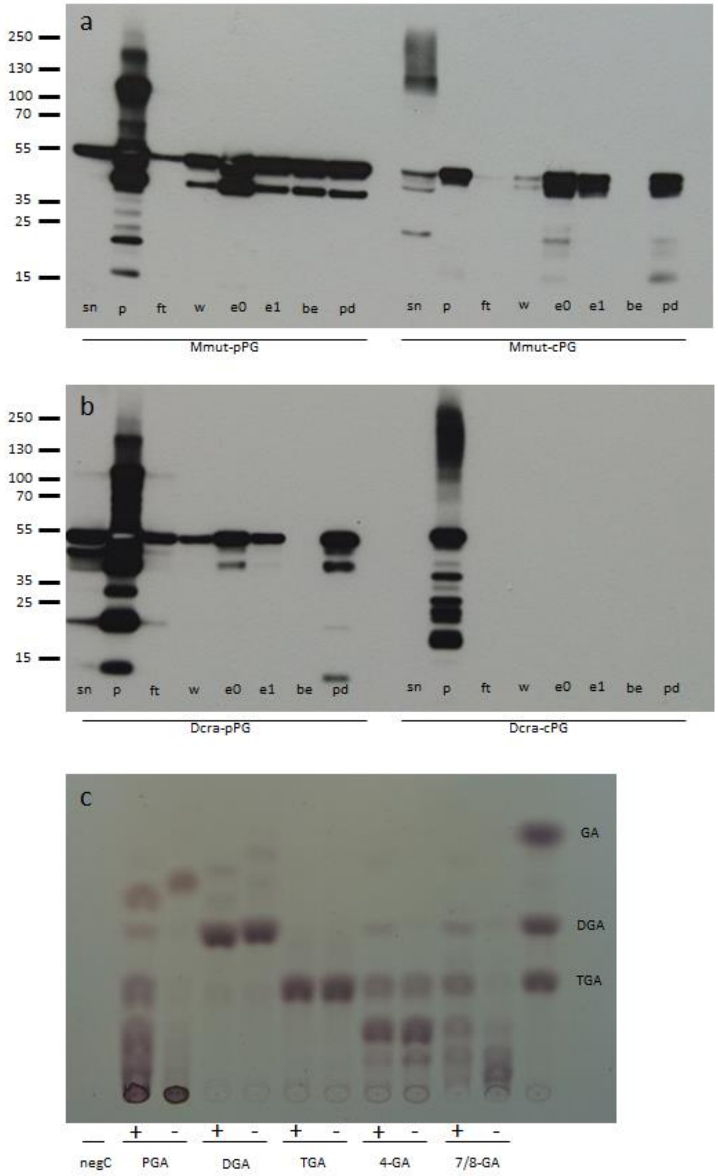

Supplementary Figure 5: Heterologous expression and functional characterization of GH28 proteins from Donaciinae symbionts. (a-b) Western Blot of recombinant plasmid- and chromosome-encoded GH28s from (a) *M. mutica* and (b) *D. crassipes* symbionts. Major bands at about 50 kDa correspond to target proteins and each lane corresponds to one fraction in the purification process (sn: supernatant after cell lysis, p: pellet after cell lysis, ft: flow-through from IMAC of sn, w: wash from IMAC, e0: first elution; e1: second elution be: e0 after buffer exchange (elution buffer to assay buffer), pd: pull down of e1 (anti-V5 agarose beads). The molecular weight standard is shown on the left hand side in kDa. Note that Dcra-cPG formed only inclusion bodies and that Mmut-cPG and Dcra-pPG were lost after buffer exchange. (c) Thin-layer chromatography assessing the efficiency of Mmut-pPG in breaking down different pectic substrates. Substrates: PGA: polygalacturonic acid, deesterified (no methylation); DGA: digalacturonic acid, TGA: digalacturonic acid, 4-GA: tetragalacturonic acid, 7/8-GA: mixture of hepta- and octamer of galacturonic acid. NegC: negative control (no sample). Each substrate was either incubated with (+) or without (-) the Mmut-pPG. Source data are provided as a Source Data file.

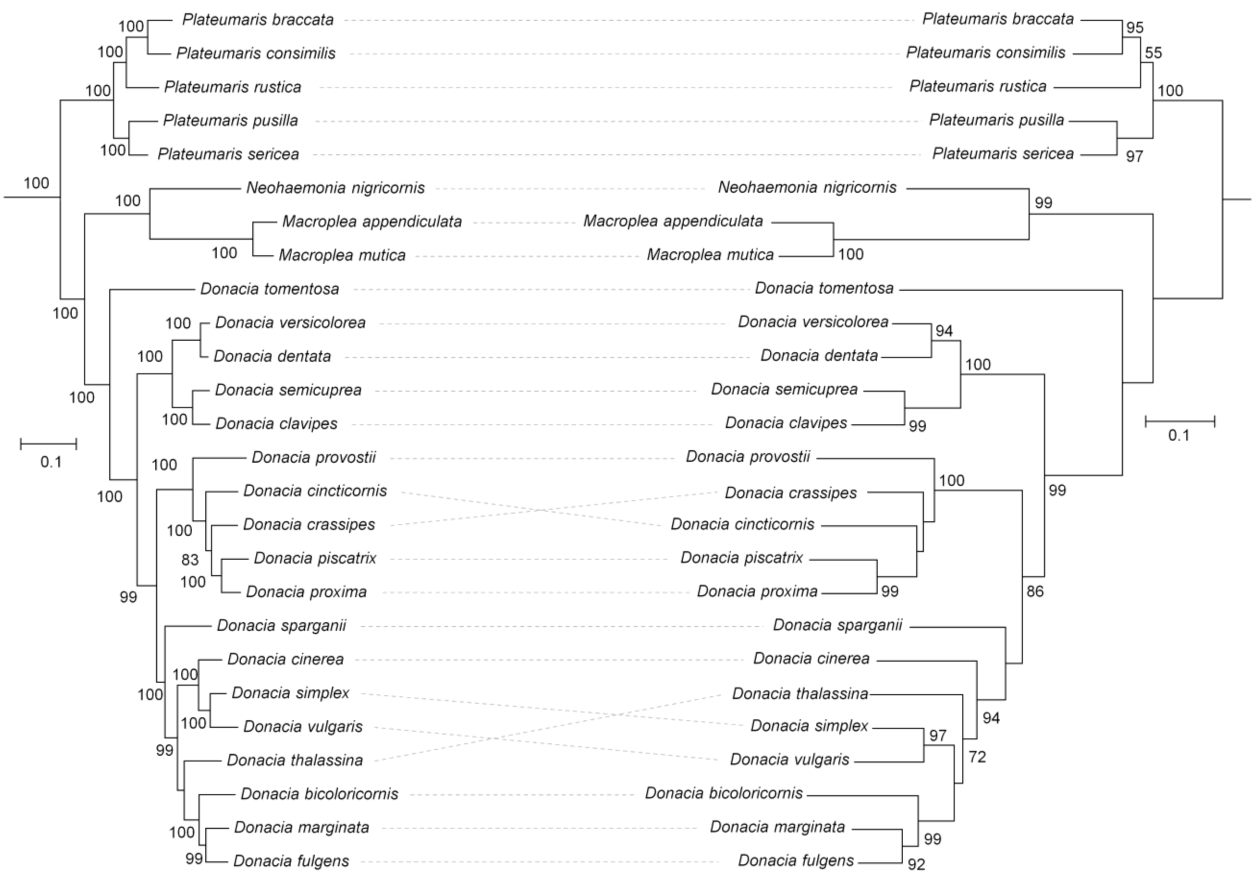

Supplementary Figure 6: Phylogeny of plasmid-encoded *trfA* mirrors that of the entire symbiont genome. Left side: Phylogenomic tree of Donaciinae symbionts based on 49 marker genes (FastTree analysis implemented in Kbase; local support values are given at the nodes). Right side: Phylogeny of the plasmid-encoded gene trfA (FastTree analysis; local support values are given at the nodes). Note that the two discrepancies between trfA and phylogenomic tree are statistically not supported in the trfA phylogeny.

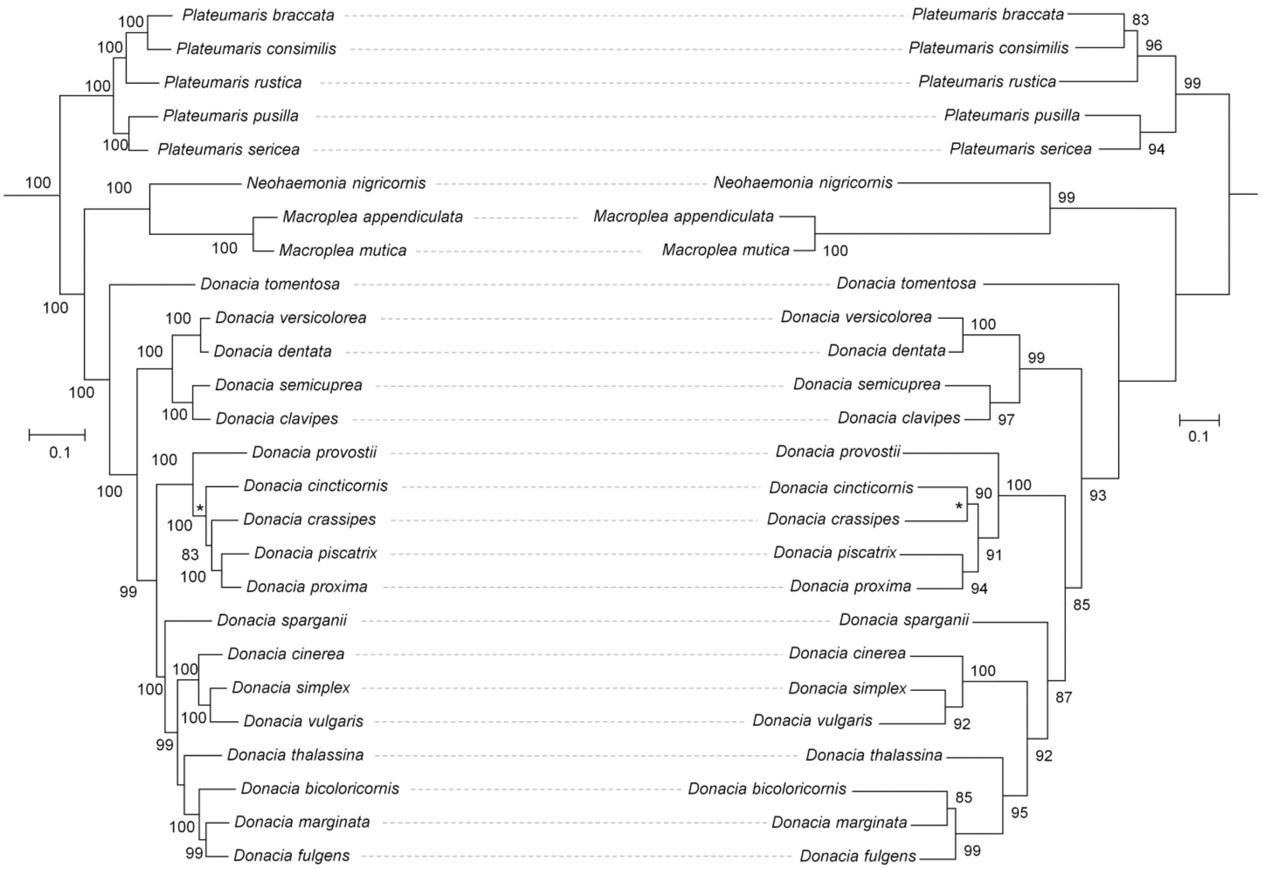

Supplementary Figure 7: Phylogeny of plasmid-encoded bifunctional chorismate mutase/prephenate dehydratase mirrors that of the entire symbiont genome. Left side: Phylogenomic tree of Donaciinae symbionts based on 49 marker genes (FastTree analysis implemented in Kbase; local support values are given at the nodes). Right side: Phylogeny of the plasmid-encoded gene annotated as bifunctional chorismate mutase/prephenate dehydratase (FastTree analysis; local support values are given at the nodes). The asterisks denote a discrepancy between both phylogenies. Note that the same discrepancy is also observed between host and symbiont phylogenies (Fig. 6).

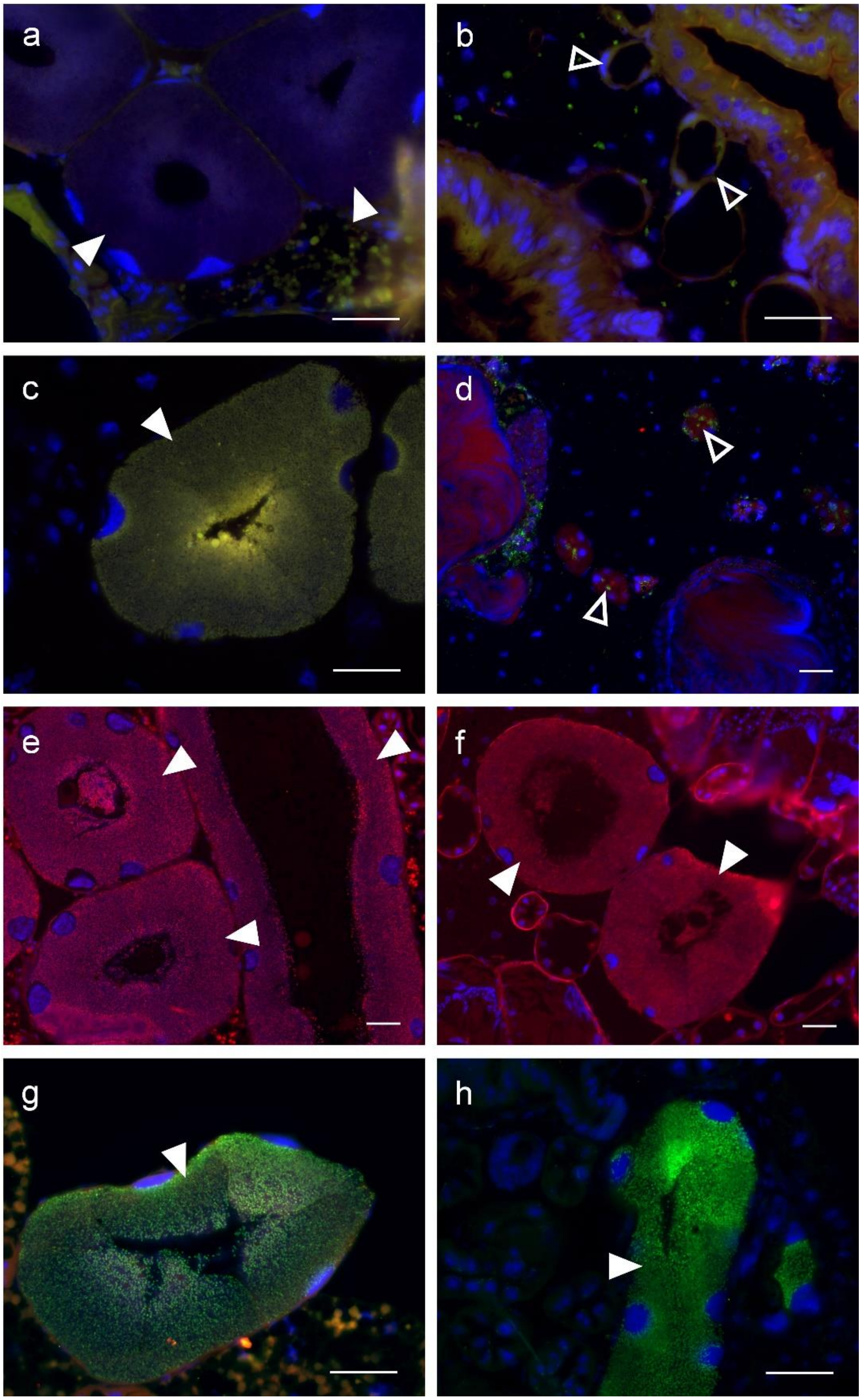
a
b
c
d
e
f
g
h

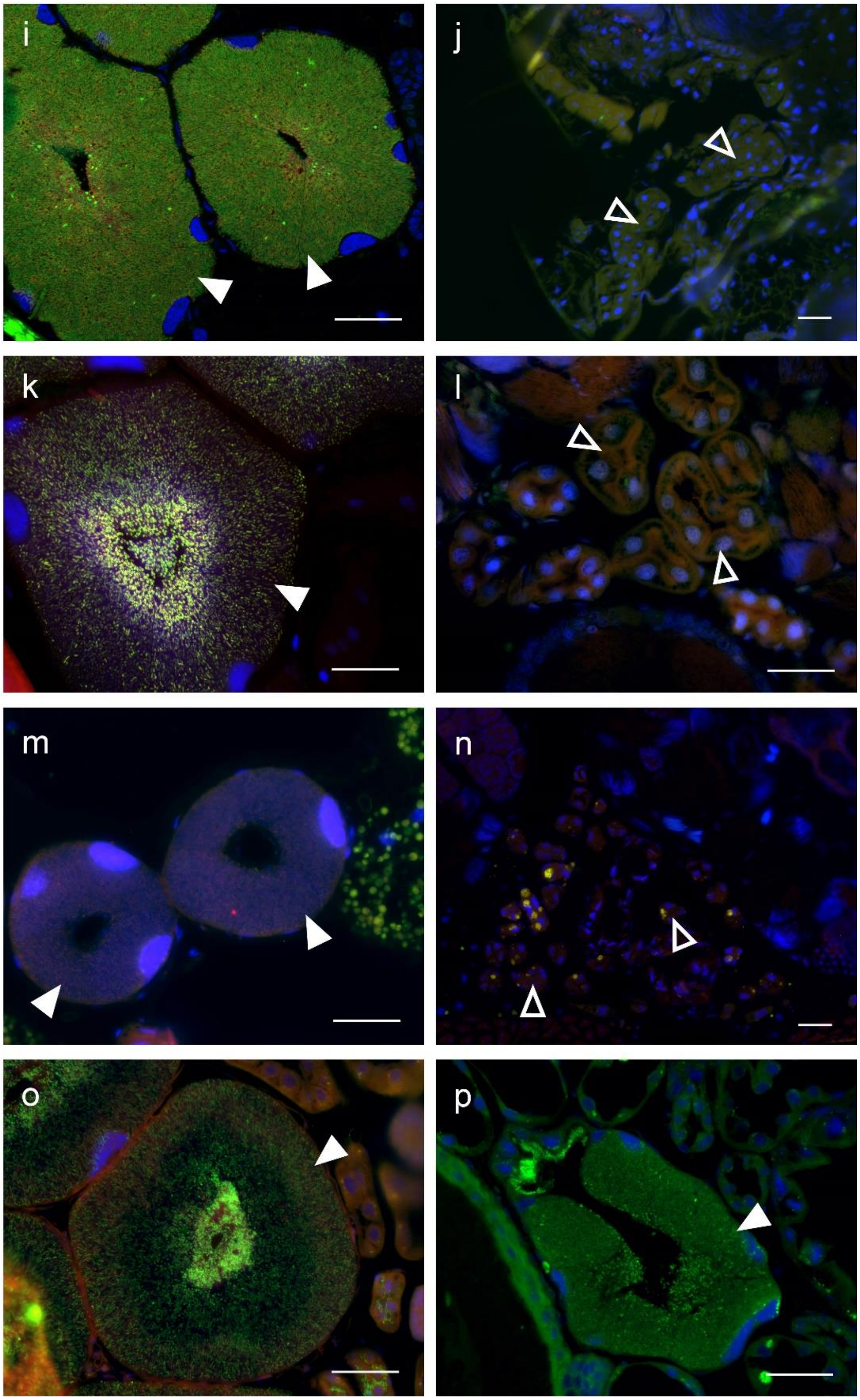
i
j
k
l
m
n
o
p

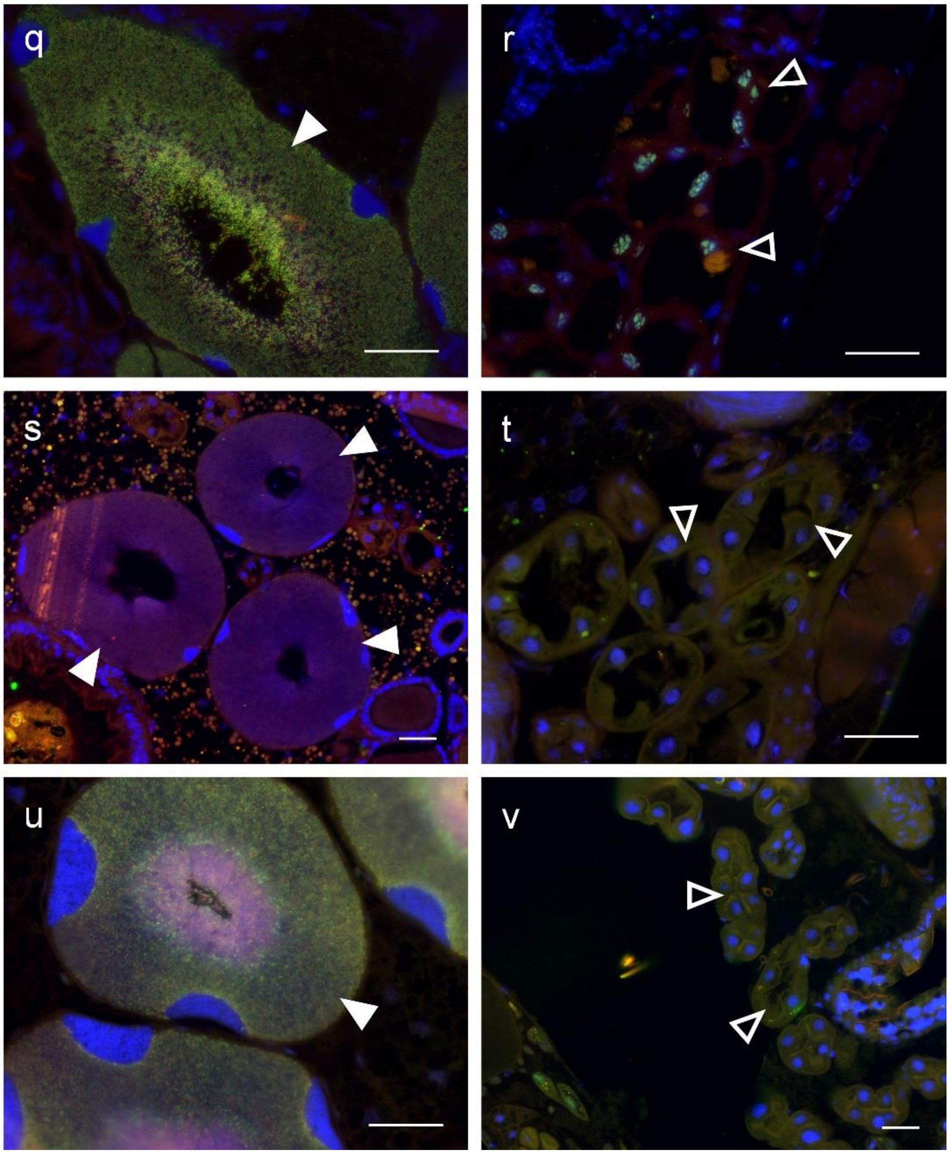

Supplementary Figure 8: Fluorescence in situ micrographs of symbiotic organs in female (left panel) and male (right panel) reed beetles. Note that different probes were used for the different species (Cy3- and Cy5-labeled, see Supplementary Table 2), and for some species, rRNA quality was not sufficient to result in successful staining, so bacterial symbionts are only labeled by DAPI (blue) (see **a**, **m**, and **s**). Filled white arrowheads highlight symbiont-filled Malpighian tubules (symbiotic organs), empty arrowheads point to Malpighian tubules without symbionts (but occasionally other bacteria unspecifically infect the Malpighian tubules as well as other tissues, see **d** and **r**). The following species were tested (host plant order given in brackets): (**a-b**) *Donacia cinerea* (Poales), (**c-d**) *Donacia clavipes* (Poales), (**e-f**) *Donacia crassipes* (Nymphaeales), (**g-h**) *Donacia dentata* (Alismatales), (**i-j**) *Donacia semicuprea* (Poales), (**k-l**) *Donacia simplex* (Poales), (**m-n**) *Donacia thalassina* (Poales), (**o-p**) *Donacia versicolorea* (Alismatales), (**q-r**) *Donacia vulgaris* (Poales), (**s-t**) *Plateumaris consimilis* (Poales), (**u-v**) *Plateumaris sericea* (Poales). Note that only the Alismatales- and Nymphaeales-feeding species show symbiont-bearing organs in adult males (**f, h, p**), whereas the females of all species carry symbionts. Scale bars 50 µm.
