# Supplementary material for: Bacterial symbionts support larval sap feeding and adult folivory in (semi-)aquatic reed beetles
Source: Nat Commun. 2020 Jun 11;11:2964. doi: 10.1038/s41467-020-16687-7 (PMC7289800; doi:10.1038/s41467-020-16687-7)
Supplement: Supplementary file 3 — Description of Additional Supplementary Files [file 41467_2020_16687_MOESM3_ESM.pdf]

Description of Additional Supplementary Files

File Name: Supplementary Data 1

Description: OthoMCL table of orthologous gene clusters across the symbiont genomes of 26 Donaciinae host species in four genera. Given are the cluster numbers (derived from kbase OrthoMCL analysis), representative function, KO hierarchy annotation (level 1-3), and the presence and location of the CDS across the 26 genomes (number indicating the position of the CDS in reference to *greA*). For better readability, adjacent orthologs with the same annotation were combined (assuming that they represent artifacts of split CDS due to sequencing errors), and orthologous clusters without annotation or annotated as hypothetical proteins that are present in only one genome were removed. Note that the gene annotated as Cystathionine gamma-synthase (EC 2.5.1.48, metB) was fragmented and likely pseudogenized in both Dcla and Dsem.

File Name: Supplementary Data 2

Description: Summary of the distribution of plant cell wall degrading enzymes in Donaciinae adult gut transcriptomes (14 species). Given are the numbers of GH9, GH45, and GH48 copies per transcriptome, as well as for each transcriptome the detection of different GH copies when using various sequencing depths.

File Name: Supplementary Data 3

Description: Identification of genes encoding carbohydrate-active enzymes in Donaciinae adult gut transcriptomes, using dbCAN (http://bcb.unl.edu/dbCAN2/index.php) a web server for automated carbohydrate-active enzyme annotation. Note that in the case of *Donacia crassipes, D. versicolorea* and *Macroplea appendiculata*, dbCAN also identified sequences encoding GH28 proteins, which correspond to the symbiont-derived pectinases.

File Name: Supplementary Data 4

Description: Sequences of the codon-optimized pectinase genes from the symbiont genomes of *Donacia crassipes* and *Macroplea mutica* used for heterologous expression.
