# Supplementary material for: Bacterial symbionts support larval sap feeding and adult folivory in (semi-)aquatic reed beetles
Source: Nat Commun. 2020 Jun 11;11:2964. doi: 10.1038/s41467-020-16687-7 (PMC7289800; doi:10.1038/s41467-020-16687-7)
Supplement: Supplementary file 7 — Supplementary Data 4 [file 41467_2020_16687_MOESM7_ESM.zip › 240258_2_data_set_4606782_q518t2.docx]

>Dcra-pPG

AAGGACACCCGTAGCATCACCGAGCCGGTGATCCCGAAGATTTGCAAAACCCTGATTGCGAACGACAACAAAGATTTCACCAACGAAATCAACGACGCGGTTACCGATTGCGCGAAGCAGCACAAAGTGGTTAGCCTGATCAGCAGCAACAACGGCGATACCCACTTTTACAGCGGTCCGATCATTATCCCGAGCTATGGTGGCATCCTGATTAACAAGACCGTGATCCTGAGCGCGATTAACGACCCGATCCTGTACGATAAGGGCAACAAAACCTGCGGTACCCTGGACGATATTGGCAAGGGCTGCAACCCGTTCATTATCCTGAAAGGCGAGAACAGCGGTCTGTACGGTGAAGGCTATATCGACGGCCAAGGTGGCATGCTGCTGAAGAACAAGAAATATACCTGGTGGCAGCTGGCGACCGAGGCGAAAATTAGCCACAAGAAACAAAACAACCCGCACCTGATCAACATTAACAGCGGTCACAACGTGATTATCTACAACCTGCACCTGATCAACAGCCCGAACTTTCACGTGGTTCCGTATCAGACCAACGGCCTGACCATTTGGGGTGTTAAAATCAACACCCCGGCGGATGCGCGTAACACCGACGGCATTGATCCGAGCAGCAGCCAAAACATCACCATTACCCACAGCAACATCAGCACCGGTGACGATAACATCGCGATTAAGGCGGGCAAGAAAGGTGTGAGCAAACACATCACCATTATCAACAACAACTTCGGTTACGGCCACGGTATGAGCATTGGCAGCGAGATCAACAGCGGTGTTAACGATATCCTGATTAAGAACCTGACCCTGAAAAACACCACCAACGGTCTGCGTATTAAGAGCAACATCACCAAAGGTGGCGTGGTTACCAACATCCACTACGAAAACATCTGCATTTTTAACGTTAAGAACCCGATTATCCTGGACACCAACTATAACAACGATAAGGAGAACGAGGAGAAGAGCATTCCGCAGTTCAAGAACATCTTCTTTAAGAACATCGAAATCCTGACCAGCGGCCTGCTGAAATTTAACGGTCTGAACGAGGAAAACATGATCGAAATTTTCGTGGACAACTTTCACGTTAAGAACGGCAGCAGCTGGATTAAAAACCACATTATCATTCACGGTAACATCGACTACATCATTAGCAAGGATAACTGCACCCTGTATAAAGGCGAGCTGAACAGCAAGCTGGAAGGTAAACCGATCCCGAACCCGCTGCTGGGTCTGGATAGCACC

>Mmut-pPG

GAGGACCAGCGTATCGTGCACGAACCGATCATTCCGAAGATTTGCCAGGTTCTGGAGGCGAACGGTCAAGATAGCACCCTGCGTATCCAGCAAGCGATTAACTACTGCGCGCACAAACACCAGATCGTGAGCCTGATTGCGAGCAAGAACAAACAACACGTTTTCTACATCAGCCCGATCAACATTCCGGACTATGGTGGCCTGCTGATTAACCAAACCGTGACCGTTGCGGCGATCAGCAACAGCAGCCTGTTTGATATTACCAACAAGCACGAGTGCGGCAACCTGAGCATCATTAGCAGCGTGAAGTACTGCAAACCGCTGATCACCATTTATGGCAAAAACAACGGTATCTACGGTAACGGCTATATTGACGGTCAGGGTGGCGTTCTGCTGAAGAACAAGAAATACACCTGGTGGCAACTGGCGAGCGAAGCGCAGATCCAAAACAAACAGCAAAACGCGCCGCAGCTGATCAACATTAACGATGGTAACAACACCATCCTGTATAAGATCCACCTGATTAACAGCCCGAACTTCCACATTGTGAGCCACAACACCAACGGCCTGACCATCTGGGGTGTTACCATTAAAACCCCGGCGGATGCGCGTAACACCGATGGTATCGATCCGATGAGCAGCCAGAACATCACCATTGCGAACAGCTACATCAGCACCGGTGACGATAACATCGCGATTAAGGCGGGCAAACAAGGTACCAGCAAGAACATGACCATCATTAACAACGTGTTTGGTTATGGCCACGGTATGAGCATCGGCACCGAGATTCAGAGCGGTGTTAGCAACATCCTGATTAAGAACCTGAGCCTGATCGACACCACCAACGGCCTGCGTATTAAAAGCGATAGCACCCGTGGTGGCCTGGTGACCAACATCAACTTCCACAACATTTGCATGCTGAACGTGAAGAAACCGATCGTTCTGGACGCGTTTTACAACAAGAACATCCACGGTAACTACATTCCGCAGTACAAGAAAATCAACTTCAACAACATCAACGTGCTGACCCTGGGCGAATACGTTTTCAACGGTTTCAACGAGAAAAACATCATCGAAGTGTTCTTTAAGAACGTTCACATCAAACCGGGCAGCATCTGGATTAAGAAAAACGTGCACATCACCGGCTTTATTAACAACGACGCGCAAGGTGATCACTGCCCGGTTTACAAAGGCGAGCTGAACAGCAAGCTGGAAGGTAAACCGATCCCGAACCCGCTGCTGGGTCTGGATAGCACC

>Dcra-cPG

TACAACAGCAAGAAAAAGAACTATATCCTGGACCAGATCAAGATCCCGTTCTTTAACAAAAAGAAATACTTCGTGATCGAGTATATCAAATTCTTTAACAAGAAAAACGACATTAGCTACGCGATCAACAACGCGATTAGCGATTGCCACAGCAACGGTGGCGGTATCGTGATCATTCCGAACGGCGAATTCTATACCAGCACCATCATTCTGAAGAGCAACGTTAACCTGCACCTGCAAAACGACACCATCCTGAAGTTTTACACCGATCCGAACAAATACTATAACGTGTTCACCCGTTGGGAGGGTACCGAATGCATCAACTATGTTAGCCTGATTTACGCGTATAACCAGAAGAACATTGCGATCACCGGCAAAGGTACCCTGGACGGCCAAGCGAACTTCTACAACTGGTGGAGCTGGAAGAACGATATCAACGGTAACCACCTGCAGAACAACGACGTGAAGATCCTGAAGGATATGAACAAGAACAACATCCCGATCAAAAACCGTGTTTTCGGTATCAACCACTTTCTGCGTCCGAACTTCATTCAGCTGTATCTGTGCAAAAACATTTTTATCAGCGACATCAACATCATTAACAGCCCGATGTGGGAGATTAACCCGGTGCTGAGCAAGAACGTTATCATCCAAAACATCAAGATCAACAGCCTGGGCCCGAACAACGACGGTTGCAACCCGGAAAGCTGCAACAACGTGCTGATCAAGAACAACATTTTCTACACCGGCGACGATTGCATTGCGATCAAAAGCGGCACCAACAACGATGGTCGTAAGATCAACGTTCCGAGCAAAAACATCATTATCAAGAAATGCCAGATGTATAACGGCCACGGTGCGATCACCCTGGGTAGCGAGTGCAGCGGCGGTATCAAGAACATCTTCATCGAAAACTGCACCATCAACGATACCATTCAGAGCTTCTTTAAGATCAAAAACAACGCGGAGCGTGGCGGTAACATCAACAACATCTACATCAAGAACATTAACATCAAGTTCATCCAAAACAACTTCTTTAACATCAACTACCTGTATGACGAGGGCGATAAGGGTAACTTCATCCCGATCGTGAAGAACATCTTCATCAGCGACATCAACGCGGAAAACTGCCTGCAAGTTTTCAACATCAACACCTTCAAGAAAAGCATTGTGGATAACGTTTTCTTTAAGAACTGCATCTTCAAAGGCCTGAAGAAACCGGAAAAGATCCTGATCTACAACAAGTACAACATCAACATTATCAACACCAAGTTCATTAGCAAAGGCGAGCTGAACAGCAAGCTGGAAGGTAAACCGATCCCGAACCCGCTGCTGGGTCTGGATAGCACC

>Mmut-cPG

TTCAACTACAAGCAGTTTAACGCGATTCGTAAAAACATCAGCAGCATTTATACCAACCAGAAGCAACACTACATCTATACCCACCTGAAGAACAAAACCGACATCACCATCATTATCAACAACACCATTACCAACTGCCACAACAACGGTGGCGGTATTATCGTGATCCCGAACGGCGAGTACTATAGCGGTCCGATCCACCTGAAGAGCAACGTGCACATTCACCTGCAAGACGATGTTATCATCAAGTTCTACACCGACCCGAAGAAATATTTCAACGTGCTGACCCGTTGGGAAGGCATCGATTGCATTAACTACACCCCGCTGATCTACGCGTATAAGCAGAAAAACATCGCGATTACCGGCCACGGTGTTCTGGATGGCCAAGCGAGCATGAACAACTGGTGGAGCTGGAAAAACGACATTAACGGTAACTACCTGCAGAACAAGGATGTGAAAGTTCTGATCAACATGATGCGTAACAACATCCCGATTAAGAACCGTATTTTTGGTTACCACCACTATCTGCGTCCGAACTTCATCCAATTTTATCTGTGCACCAACATCCTGATTAAAAACATCACCATTATCAACAGCCCGATGTGGGAGATTCACCCGGTGCTGAGCAACAACATCCACATTAGCCACGTTCACATCAACAGCATTGGCCCGAACAACGACGGTTGCAACCCGGAGAGCTGCAACAACGTTCTGATCGAACACTGCAGCTTCAACACCGGCGACGATTGCATCGCGATTAAGAGCGGCAAAAACAACGATGGTCGTAAGAACAACATCCCGAGCAGCAACATTATCATTCAGAACTGCCTGATGTACAAAGGCCACGGTGCGGTGGTTCTGGGCAGCGAATGCAGCGGCGGTATCAACAACATCTTCATTAAGAACTGCCAGACCTTTGGTAAGAAACTGCAAAGCTTCCTGCGTATTAAAAACAACGCGGTGCGTGGCGGTAACATCTACGAGATTTATCTGCAGGACACCAAGATCTACTGCGTGAACAGCAGCATCCTGAACATTAACTTTGTTTACGATGAAGGCCAACACGGTAGCTATATCCCGAAGGCGTACAACATCTACATCAACAACATCTACGCGTACATGTGCTACCGTGTTATGGACATCAACACCTTTCAGAAGAGCTATGTGAACAACATCTTCCTGAAAAACAACGTTTTTCAGCACATTCAATACCCGAGCAAGATCAACATTTGCAACCCGAACAACATTTACCTGTTCAACACCAAGTATCTGAAAGGCGAGCTGAACAGCAAGCTGGAAGGTAAACCGATCCCGAACCCGCTGCTGGGTCTGGATAGCACC
