# Supplementary material for: Bacterial symbionts support larval sap feeding and adult folivory in (semi-)aquatic reed beetles
Source: Nat Commun. 2020 Jun 11;11:2964. doi: 10.1038/s41467-020-16687-7 (PMC7289800; doi:10.1038/s41467-020-16687-7)
Supplement: Supplementary file 8 — Reporting Summary [file 41467_2020_16687_MOESM8_ESM.pdf]

nature research

Corresponding author(s): Martin Kaltenpoth

Last updated by author(s): May 5, 2020

Reporting Summary

Nature Research wishes to improve the reproducibility of the work that we publish. This form provides structure for consistency and transparency in reporting. For further information on Nature Research policies, see Authors & Referees and the Editorial Policy Checklist .

Statistics

For all statistical analyses, confirm that the following items are present in the figure legend, table legend, main text, or Methods section.

n/a	Confirmed	
☐	☒	The exact sample size (*n*) for each experimental group/condition, given as a discrete number and unit of measurement
☐	☒	A statement on whether measurements were taken from distinct samples or whether the same sample was measured repeatedly
☒	☐	The statistical test(s) used AND whether they are one- or two-sided<br>*Only common tests should be described solely by name; describe more complex techniques in the Methods section.*
☒	☐	A description of all covariates tested
☒	☐	A description of any assumptions or corrections, such as tests of normality and adjustment for multiple comparisons
☒	☐	A full description of the statistical parameters including central tendency (e.g. means) or other basic estimates (e.g. regression coefficient) AND variation (e.g. standard deviation) or associated estimates of uncertainty (e.g. confidence intervals)
☒	☐	For null hypothesis testing, the test statistic (e.g. *F*, *t*, *r*) with confidence intervals, effect sizes, degrees of freedom and *P* value noted<br>*Give P values as exact values whenever suitable.*
☒	☐	For Bayesian analysis, information on the choice of priors and Markov chain Monte Carlo settings
☒	☐	For hierarchical and complex designs, identification of the appropriate level for tests and full reporting of outcomes
☒	☐	Estimates of effect sizes (e.g. Cohen's *d*, Pearson's *r*), indicating how they were calculated

Our web collection on statistics for biologists contains articles on many of the points above.

Software and code

Policy information about availability of computer code

Data collection	No software was used for data collection.
Data analysis	Canu, Geneious 11.0.5, KBase Web server, FastQC v1.0.4, Trimmomatic v0.36, SPAdes v3.12.0, Busybee, Kraken, RStudio version 1.1.453 (including the seqinr package), RAST v2.0, OrthoMCL v.0.0.7, BlastKOALA, OmicCircos, FastTree 2, CD-HIT Suite server, MAFFT version 7, IQ-TREE web server, ncbi_nr protein database, KEGG database, PhyML, RAxML, CLC Genomics Workbench v11.0, dbCAN2 meta server.

For manuscripts utilizing custom algorithms or software that are central to the research but not yet described in published literature, software must be made available to editors/reviewers. We strongly encourage code deposition in a community repository (e.g. GitHub). See the Nature Research guidelines for submitting code & software for further information.

Data

Policy information about availability of data

All manuscripts must include a data availability statement. This statement should provide the following information, where applicable:

- Accession codes, unique identifiers, or web links for publicly available datasets
- A list of figures that have associated raw data
- A description of any restrictions on data availability

Symbiont genome and host mitochondrial genome sequences are available in the SRA of NCBI under BioProject number PRJNA587602 [https://www.ncbi.nlm.nih.gov/bioproject/PRJNA587602], and host transcriptome sequencing data under PRJNA575113 [https://www.ncbi.nlm.nih.gov/bioproject/PRJNA575113]. The following databases were used in this study: KBase, NCBI protein database, KEGG database, CAZy database, dbCAN HMM database and Hotpep peptide database. The source data underlying Figure 5 and Supplementary Figures 3-5 are provided as a Source Data file.

Field-specific reporting

Please select the one below that is the best fit for your research. If you are not sure, read the appropriate sections before making your selection.

☒ Life sciences ☐ Behavioural & social sciences ☐ Ecological, evolutionary & environmental sciences

For a reference copy of the document with all sections, see nature.com/documents/nr-reporting-summary-flat.pdf

Life sciences study design

All studies must disclose on these points even when the disclosure is negative.

Sample size	This is a comparative genomics and transcriptomics study, using individual replicates of genomes/transcriptomes per species and comparing across a phylogenetic diversity of taxa. Phylogenetic sampling was done in a way to cover the four major genera in the subfamily Donaciinae, and include representatives of the different clades per genus, abased on a previous phylogeny of Kölsch and Pederson 2008.
Data exclusions	No data were excluded.
Replication	Symbiotic organs were dissected from 2-10 specimens per host species, and localization was consistent throughout, as represented in Figure 1d. Fluorescence in situ hybridization to localize the microbial symbionts in adult beetles' Malpighian tubules (Figure 1e, Figure 7, and Supplementary Figure 8) was performed on one (Donacia cinerea; Donacia clavipes; Donacia crassipes; Donacia dentata; Donacia semicuprea; Donacia simplex; Donacia thalassina; Donacia vulgaris male; Plateumaris sericea) or two (Donacia versicolorea; Donacia vulgaris females; Plateumaris consimilis) specimens per species and sex, yielding consistent results. The heterologous expression of GH28 proteins (Supplementary Figure 5) was performed three times. The success of heterologous expression and subsequent Immobilized Metal Affinity Chromatography was monitored three times but the pull down using anti-V5 agarose beads was just performed and monitored once. Replicated experiments yielded consistent results.
Randomization	Randomization was not performed, as the order of samples was deemed irrelevant in in vitro enzymatic assays and sequencing experiments.
Blinding	The outcome of in vitro enzymatic assays and sequencing experiments could not be systematically affected by the experimentators, so blinding was not done.

Reporting for specific materials, systems and methods

We require information from authors about some types of materials, experimental systems and methods used in many studies. Here, indicate whether each material, system or method listed is relevant to your study. If you are not sure if a list item applies to your research, read the appropriate section before selecting a response.

Materials & experimental systems

n/a	Involved in the study	
☐	☒	Antibodies
☒	☐	Eukaryotic cell lines
☒	☐	Palaeontology
☐	☒	Animals and other organisms
☒	☐	Human research participants
☒	☐	Clinical data

Methods

n/a	Involved in the study	
☒	☐	ChIP-seq
☒	☐	Flow cytometry
☒	☐	MRI-based neuroimaging

Antibodies

Antibodies used	The horseradish peroxidase (HRP) coupled V5 tag monoclonal antibody was used in a 1:10,000 dilution.
Validation	Thermo Fisher Scientific, Waltham, MA, USA

Animals and other organisms

Policy information about studies involving animals; ARRIVE guidelines recommended for reporting animal research

Laboratory animals	No laboratory animals were used in this study.
Wild animals	Male and female adult beetles of the species Donacia bicoloricornis, Donacia cincticornis, Donacia cinerea, Donacia clavipes, Donacia crassipes, Donacia dentata, Donacia fulgens, Donacia marginata, Donacia piscatrix, Donacia provostii, Donacia proxima, Donacia semicuprea, Donacia simplex, Donacia sparganii, Donacia thalassina, Donacia tomentosa, Donacia versicolorea, Donacia vulgaris, Macroplea appendiculata, Macroplea mutica, Neohaemonia nigricornis, Plateumaris braccata, Plateumaris consimilis, Plateumaris rustica, Plateumaris sericea, and Plateumaris pusilla were collected by hand in the field, in non-protected areas

	according to the country-specific regulations After return to the laboratory, beetles were killed by freezing and subsequently dissected for further experiments.
Field-collected samples	For the transport from field to laboratory, beetles were kept in containers with fresh air supply and food plants for nutrition.
Ethics oversight	No ethical approval was necessary for field collecting of non-protected invertebrate specimens.

Note that full information on the approval of the study protocol must also be provided in the manuscript.
