# Supplementary material for: Bacterial symbionts support larval sap feeding and adult folivory in (semi-)aquatic reed beetles
Source: Nat Commun. 2020 Jun 11;11:2964. doi: 10.1038/s41467-020-16687-7 (PMC7289800; doi:10.1038/s41467-020-16687-7)
Supplement: Supplementary file 2 — Peer Review File [file 41467_2020_16687_MOESM2_ESM.pdf]

Editorial Note: This manuscript has been previously reviewed at another journal that is not operating a transparent peer review scheme. This document only contains reviewer comments and rebuttal letters for versions considered at *Nature Communications.*

REVIEWERS' COMMENTS:

Reviewer #1 (Remarks to the Author):

I have read the revised paper and think it is now acceptable.

Reviewer #3 (Remarks to the Author):

I would like to thank the authors for their detailed replies to my remarks. I have nothing else to add.
